# The interactome of multifunctional HAX1 protein suggests its role in the regulation of energy metabolism, de-aggregation, cytoskeleton organization and RNA-processing

**DOI:** 10.1042/BSR20203094

**Published:** 2020-11-13

**Authors:** Maciej Wakula, Anna Balcerak, Tymon Rubel, Mateusz Chmielarczyk, Ryszard Konopinski, Filip Lyczek, Ewa A. Grzybowska

**Affiliations:** 1 Maria Sklodowska-Curie National Research Institute of Oncology, Roentgena 5, 02-781, Warsaw, Poland; 2 Institute of Radioelectronics and Multimedia Technology, Warsaw University of Technology, 00-665, Warsaw, Poland; 3 Warsaw University, Department of Biology, Miecznikowa 1, 02-096, Warsaw, Poland

**Keywords:** HAX1, protein aggregation, protein-protein interactions

## Abstract

HCLS1-associated protein X-1 (HAX1) is a multifunctional protein involved in many cellular processes, including apoptosis, cell migration and calcium homeostasis, but its mode of action still remains obscure. Multiple HAX1 protein partners have been identified, but they are involved in many distinct pathways, form different complexes and do not constitute a coherent group. By characterizing HAX1 protein interactome using targeted approach, we attempt to explain HAX1 multiple functions and its role in the cell. Presented analyses indicate that HAX1 interacts weakly with a wide spectrum of proteins and its interactome tends to be cell-specific, which conforms to a profile of intrinsically disordered protein (IDP). Moreover, we have identified a mitochondrial subset of HAX1 protein partners and preliminarily characterized its involvement in the cellular response to oxidative stress and aggregation.

## Introduction

HAX1 protein (HCLS1-associated protein X-1) is described as an anti-apoptotic factor involved in the regulation of cell migration and calcium homeostasis [1]. Its overexpression was reported in psoriasis [2] and several neoplasms [3–6], including breast cancer, in which it was also proposed to affect metastasis [4,7]. On the other hand, mutations inactivating *HAX1* result in severe neutropenia (Kostmann disease) [8]. Interestingly, Kostmann disease has mixed etiology and may be caused by *HAX1* mutations or mutations in *ELA-2* (encoding elastase) or other genes. When *HAX1* is mutated in the region present in all splice variants (usually Q190X), in addition to neutropenia symptoms include neurological abnormalities [9]. Notably, in patients bearing W44X *HAX1* mutation in the region which undergoes alternative splicing, the main transcript variant is non-functional, but the variant in which the mutation is spliced out persists, resulting in neutropenia but without neurological symptoms [9]. These results point to some yet undefined role of HAX1 in central nervous system (CNS).

To date, 40 proteins have been specifically described to interact with HAX1, including cellular proteins and a subset of viral proteins (Supplementary File S1), and many more proteins are predicted to interact with HAX1, based on high-throughput interaction mapping data (https://targetexplorer.ingenuity.com/index.htm). While the number of proven and potential HAX1-interacting protein partners is relatively large, these proteins cannot be easily assigned to any coherent pathway or ontology group. Accordingly, HAX1 has been implicated in numerous processes and activities, including the regulation of apoptosis [10–12], cell migration [13–15], regulation of calcium concentration in the endoplasmic reticulum [16,17], actin cytoskeleton reorganization [18], granulocyte differentiation [19,20], B-cell signaling [21,22], endocytosis [15], adhesion [14,23] and ubiquitination [24]. It was also detected not only in the mitochondria, which represents its predominant localization [25,26], but also in the endoplasmic reticulum [16], on the leading edge of lamellipodia [27], in P-bodies [28] and in the nucleus [29,30]. Adding to the complexity of the role of HAX1 in the cell, this protein was demonstrated to bind mRNA and influence its stability [29–31]. Several molecular mechanisms explaining particular HAX1 molecular functions were proposed [1], but the comprehensive analysis addressing its multiple activities is still lacking.

It is worth noting, that HAX1 sequence does not display any significant homology to other proteins and it lacks any known functional domains. It was suggested that HAX1 has a region with homology to domains present in Bcl2 family of proteins [32], which would indicate its role in apoptosis, but the existence of this homology has been questioned [33].

Additionally, there is no information concerning HAX1 structure. The existence of some structural motifs was reported in relation to calcium concentration [26], but it was also proposed that HAX1 is mostly intrinsically disordered [34,35]. Disordered structure is often associated with promiscuous protein binding and may explain large number of HAX1 protein partners.

The role of HAX1 in etiology of different pathologies, including cancer, and its elusive mode of action provides reasons for the comprehensive analysis of HAX1 interactome. Establishing a full scope of HAX1 interactions would help to explain its multiple functions in connection to specific pathways, and, possibly, shed some light into its mode of binding.

In this report, we present the results of the two different approaches to obtain more comprehensive information concerning HAX1 interactome data. Using yeast two-hybrid screen (Y2H), we have analyzed human cDNA libraries from several cancers. Alternatively, HAX1-affinity purification with subsequent mass spectrometry was performed for the two cell lines derived from human cervical carcinoma (HeLa) and breast cancer (MCF7). Several potential interactions obtained in these screens were verified by co-immunoprecipitation and functional analysis. Comprehensive analysis of the obtained results indicates that, depending on the cell line, HAX1 is implicated in the regulation of cellular respiration, oxidative phosphorylation, RNA processing and cytoskeleton organization. These results are partially in line with previously reported data, but with a shift to its role in metabolism and energy-generating processes.

## Materials and methods

### Cell culture and transfections

HeLa (ATCC) and MCF7 (ATCC) cells were cultured in DMEM supplemented with 10% fetal bovine serum (Thermo Fisher Scientific). Cells were transfected with Lipofectamine (Invitrogen), according to the manufacturer’s instructions. Both cell lines were authenticated by Eurofins Genomics (Germany).

### Generation of HeLa *HAX1* KO cell line

Knockout was generated in HeLa cells using CRISPR-Cas9 system (Horizon, U.K.). RNA guide sequence: 5′ TCGAGATGAAGATGATGATG 3′. Cells were sorted on BD FACSAria III, colonies derived from single cells characterized by green fluorescence were isolated. The level of HAX1 protein was assessed by Western blot.

### Y2H

An automated high-throughput yeast two-hybrid research system from Myrexis (Myrexis, Inc., 305 Chipeta way, Salt Lake City, UT, U.S.A.) was used for the identification of direct protein–protein interactions (PPIs), with a lower rate of false-positive findings. A total of 27 baits for HAX1 were screened and gave rise to specific interactions. HAX1 also came out as a prey from 21 different baits (41 baits, including repeats). Nine different libraries were searched in the screens (brain, brain_substantia nigra, hypothalamus, hippocampus, spleen, liver, colon, testis, breast cancer/prostate cancer).

### Affinity purification and mass spectrometry

#### Sample preparation for proteome analysis

Cells were washed with phosphate-buffered saline solution (PBS), detergent permeabilized (lysis buffer: 150 mM NaCl, 20 mM Tris/HCl pH 7.4, 0.75% Nonidet-P40, supplemented with Complete Protease Inhibitor Cocktail, Roche 05056489001) and harvested using cell scraper. Cell lysate was centrifuged and supernatant was subjected to immunoaffinity chromatography via magnetic bead extraction by using anti-HAX1 rabbit polyclonal antibody (Thermo Fisher Scientific) or, for negative control, anti-IgG rabbit polyclonal antibody (Cell Signaling), coupled to Dynabeads protein A (1.5 mg, Thermo Fisher Scientific). Immunoprecipitation was carried out overnight, at 4°C, with constant agitation. Beads with antigen–antibody complexes were collected using magnetic separation method. Proteins bound to beads were suspended in 50 μl of 100 mM NH_4_HCO_3_. A total of 0.5 M TCEP (Tris-(2-carboxyethyl) phosphine (Sigma–Aldrich)) was added to the sample for a final concentration of 5 mM and incubated at 60°C for 1 h. To block reduced cysteine residues, 200 mM MMTS (S-Methyl methanethiosulfonate (Sigma–Aldrich)) was added at a final concentration of 10 mM, and the sample was subjected to incubation at room temperature for 10 min. For digestion, trypsin (Promega) was added at a 1:20 vol./vol. ratio (1 μg of trypsin per 2.5–36.3 μg of the protein sample, depending on the sample) and the sample was incubated at 37°C overnight. Finally, trifluoroacetic acid was used to inactivate trypsin. Resulting peptides were analyzed by LC-MS/MS for peptide identification.

#### MS settings

LC-MS/MS measurements were performed on a QExactive hybrid quadrupole orbitrap mass spectrometer (Thermo Scientific) coupled with a nanoAcquity LC system (Waters). Data-dependent MS to MS/MS switch mode was used for acquisition, and peptide fragmentation was achieved by high-energy collision dissociation (HCD). Spectrometer parameters were as follows: polarity mode, positive; capillary voltage, 2 kV. A sample was first applied to the nanoACQUITY UPLC Trapping Column (Waters) using water containing 0.1% formic acid as the mobile phase. Next, the peptide mixture was transferred to the nanoACQUITY UPLC BEH C18 Column (Waters, 75-μm inner diameter; 250 mm long) and an ACN gradient (5–30% over 120-min) was applied in the presence of 0.1% formic acid with a flow rate of 250 nl/min and eluted directly to the ion source of the mass spectrometer. Each LC run was preceded by a blank run in order to avoid sample carryover between the analyses. Proteomic data presented in this work were performed in the Laboratory of Mass Spectrometry at IBB PAS (Warsaw).

#### MS data processing

The acquired MS/MS raw data files were processed to peak lists with Mascot Distiller (version 2.5.1, Matrix Science) and submitted to the Mascot search engine (version 2.5.1, Matrix Science). The database was composed of SwissProt (release 2017.04) *Homo sapiens* protein records, SwissProt *Oryctolagus cuniculus* IgG entries, and contaminant proteins retrieved from the common Repository of Adventitious Proteins (cRAP, http://www.thegpm.org/crap/index.html). In total, the database included 20278 target sequences, and the same number of reversed decoy records. The search parameters were as follows: enzyme specificity: semitrypsin; maximum number of missed cleavages: 1; protein mass: unrestricted; parent ions mass error tolerance: ±5 ppm after recalibration; fragment ions mass error tolerance: ±0.01 Da after recalibration; fixed modifications: Methylthio C (45.98772 Da); variable modifications: Oxidation M (15.99492 Da). The statistical significance of identified peptides was determined using a target/decoy database search approach and a previously described procedure that provided *q*-value estimates for each peptide spectrum match (PSM) in the dataset [36,37]. Only PSMs with *q*-values ≤0.01 were regarded as confidently identified. Furthermore, all the peptide sequences matched with database entries representing contaminant proteins were rejected. Proteins represented by less than two peptides were excluded from further analysis. Proteins identified by a subset of peptides from other proteins were filtered out from the results, and those matching the same set of peptides were grouped together into clusters (metaproteins). Mascot results processing was performed using MScan, a software tool available at http://proteom.ibb.waw.pl/mscan.

The mass spectrometry proteomics data have been deposited in the ProteomeXchange Consortium via the PRIDE [38] partner repository with the dataset identifiers, PXD007887 and PXD007888.

### Immunoprecipitations and Western blotting

#### Cloning

cDNA of the selected potential interaction partners identified in the two-hybrid screen and mass spectrometry (caseinolytic peptidase B protein homolog (CLPB), SLC25A11, SLC25A12, SLC25A13, CRYAB, HRAS, PDGFB, TRIP-10, SEPT7, SET) were PCR-amplified using high-fidelity polymerase (Phusion, Thermo Fisher Scientific). Primer sets and general cloning strategy for the constructs are presented in Supplementary Table S1. The expression vectors with Aurora A kinase (AURKA) and TRIM25 cDNA were obtained from Dr Stefano Ferrari (University of Zurich) and Dr Gracjan Michlewski (University of Edinburgh), respectively. The coding sequence of human DDX3X was obtained from Prof. Robin Reed (Department of Cell Biology, Harvard Medical School, Boston, U.S.A.), modified (the hemagglutinin tag was removed from the C-end, the duplicated FLAG tag added to the N-terminus and the S588 residue was restored) and subcloned into pCR3 expression plasmid vector (Invitrogen), using HindIII/EcoRI cloning sites. SQSTM1 cDNA was re-cloned from the Addgene vector HA-p62 addgene-plasmid-28027-sequence-115905 into pEGFP_N2 using sites EcoRI/ PstI.

#### Immunoprecipitation

HeLa cells were transfected with constructs encoding selected proteins, along with the construct encoding tagged HAX1. Cells were harvested for immunoprecipitation 24 h following transfection, lysed in NP-40 lysis buffer (10 mM Tris/Cl pH 7.5, 150 mM NaCl, 0.5 mM EDTA, 0.5% Nonidet P40) supplemented with Complete Protease Inhibitor Cocktail (Roche, 05056489001). After preincubation with protein A-coated agarose beads (Dynabeads, Invitrogen), equal amounts of protein (200 μg) from cell lysates were immunoprecipited with 40 μl of anti-FLAG M2 antibody beads (Sigma), anti-c-Myc Magnetic Beads (Pierce) or GFP-trap (Chromotek).

#### Western blotting

The immune complexes were heat-denaturated in loading buffer buffer (50 mM Tris/HCl, 0.01% Bromophenol Blue, 1.75% 2-mercaptoethanol, 11% glycerol, 2% SDS) and analyzed for the presence of tagged proteins by Western blot as in Grzybowska et al., 2013 [30]. Briefly, after separation by 10–12% SDS/PAGE, the complexes were transferred on to Immobilon-P PVDF membrane (Merck Millipore, MA, U.S.A.; cat. IPVH00010), blocked for 45 min using 5% low-fat milk solution and incubated overnight at 4°C in blocking solution containing one of the following antibodies: 1:2000 anti-FLAG M2 HRP-conjugated (Sigma, MO, U.S.A.; cat. A8592), 1:1000 rabbit 27H5 anti-RAS (Cell Signaling Technology, MA, U.S.A.; cat. 3339), 1:1000 mouse GF28R anti-GFP (Thermo Fisher Scientific, MA, U.S.A.; cat. MA5-15256), 1:1000 rabbit anti-HAX1 (Thermo Fisher Scientific, MA, U.S.A.; cat. PA5-27592). Rabbit antiobody for Aurora A (1:500) was obtained from Dr Stefano Ferrari (University of Zurich). After washing, membranes were incubated 1–3 h room temperature with the appropriate HRP-conjugated secondary antibody: goat anti-rabbit IgG (1:5000, Abcam, GB; cat. 97051) or goat anti-mouse IgG (1:10000, Abcam, GB; cat. ab97023). Washed membranes were then developed using HRP detection kit WesternBright Quantum (Advansta, CA, U.S.A.; cat. K-12042).

### Detection of aggregates

Protein aggregates were visualized using Proteostat (Enzo Life Sciences) fluorescent dye, according to manufacturer’s instructions. Briefly, cells were fixed using 4% formaldehyde, permeabilized with 0.5% Triton X-100, 3 mM EDTA solution and incubated with Proteostat dye for 30 min Images of cells stained with Proteostat and Hoechst 33342 (nuclei) were obtained on Zeiss Axio observer Z1 LSM 800 confocal microscope with Plan-Apochromat 63×. Fluorescence of each dye were acquired in separate channel. Gray scale images of cells stained with Proteostat dye were quantified using ImageJ software [39]. File names were encrypted (Blind Analysis Tool) and the background was subtracted using rolling ball algorithm. Aggregates were quantified using analyze particles ImageJ built-in function. Statistical significance of the aggregation assay was assessed by Mann–Whitney U-test.

### MTT assay

A total of 5 × 10^3^ HeLa cells were treated with increasing H_2_O_2_ concentrations (0, 350, 450, 550, 650, 750 μM) for 24 h (four replicates for each condition). Cells were incubated with MTT (0.5 μg/μl) for 2 h, formazan crystals were dissolved in 100 μl of DMSO and incubated for 1 h. The absorbance at 540 nm was measured using Victor 3 (PerkinElmer) plate reader. Mean background value (no cells control) was subtracted and each reading for the treated samples was normalized to the mean value for control conditions. Normalized data from all independent experiments (four experiments in four technical repeats each) were used to generate log-logistic model in R *drc* package [40]. Data visualization was done using *ggplot2* R package. Statistical significance of MTT assay was assessed by Welch’s two sample *t-*test.

### Data analyses

Data were analyzed using biological databases and web resources for functional enrichment analysis of the PPI networks: STRING (Search Tool for the Retrieval of Interacting Genes/Protein, version 11.0) [41], Enrichr (Ontologies, Biological Process 2018) [42] and the Reactome pathway databases [43]. Gene Ontology enrichment analysis included biological process, molecular function and cellular components. For the RNA-binding prediction SONAR (available at https://github.com/YeoLab/SONAR) [44], RNApred (Amino Acid Composition Based) [45] and catRapid [46] analyses were performed. ROC curve analysis was carried out to determine the overall test performance (AUC) and to calculate possible cutoff point for RBP classification score (RCS) score. Optimal cut-off value was calculated using the nearest to (0,1) method and the maximum value of the Youden index. Violin plots were generated using *ggplot2* R package.

## Results

### Database analyses suggest that HAX1 interactome is incomplete

Hithertho, 40 HAX1 interactions with other proteins have been published, with interactions obtained mostly by Y2H, often confirmed by GST-pulldown and/or immunoprecipitation. The full list of published, validated HAX1 interactions are presented in the Supplementary File S1. Six of these interactions represent viral proteins, others represent a broad range of proteins involved in apoptosis, cell migration, cytoskeleton regulation, calcium homeostasis, immune response, endocytosis, adhesion, transcription, nuclear export, extracellular matrix modification and ubiquitination. KEGG pathway analysis of this group revealed enrichments in genes involved in focal adhesion, apoptosis and cancer (Supplementary File S1), but the dataset is small and there are no obvious connections between the pathways. More potential interactions can be predicted by database analysis. Ingenuity Target Explorer (QIAGEN, Inc., https://targetexplorer.ingenuity.com/) reveals 195 proteins potentially interacting or linked to HAX1 and BioGRID (Biological General Repository for Interaction Datasets, 3.5, https://thebiogrid.org) predicts 198 interactions. The two datasets display a substantial overlap of 164 proteins. Sixty-three of these proteins were obtained by Huttlin et al. [47] using HEK293 embryonic kidney cell line with neuronal characteristics. This probably explains why gene ontology analysis of this dataset indicates significant enrichment in proteins associated with neuron development and differentiation, synaptic vesicles and neurotransmitter secretion (Supplementary File S2). However, these predictions, without further validation cannot be treated as completely reliable and do not substantially improve or clarify our knowledge concerning HAX1 interactome and its role in the cell. Interestingly, these protein sets contain mostly cytoplasmic, vesicular or extracellular proteins, which additionally indicates that it cannot be complete representation of HAX1 interactome, since this protein is mostly mitochondrial.

### Identification of HAX1 binding partners by Y2H

The Y2H screen (Myrexis) produced 37 potential new HAX1 binding partners (15 with HAX1 as a bait and 22 with HAX1 as a prey, Supplementary File S3). When HAX1 was a bait, the binding region encompassed C-terminus (150–279 aa). When HAX1 was prey in the majority of cases the binding region also encompassed C-terminus or the whole protein (Supplementary Figure S1). Only in one case (CLPB) the binding region constituted the N-terminal and the middle part of HAX1 (the smallest overlapping part from five hits: 60–139 aa).

Due to a small number of proteins in the dataset, ontology analysis is unreliable. However, domain search revealed a relatively high proportion of proteins with ankyrin repeats (ANK2, ANK3, ANKRD1, CLPB, CRYAB, RAI14) and other proteins with domains associated with actin and spectrin cytoskeleton (EPB41L2, EPB41L3, KANK1, KALRN, PDZK8, DAAM1, TRIP10, STAM2) (Supplementary Figure S2).

Most of the proteins detected in Y2H were cytoplasmic or nuclear, only one (CLPB) was exclusively mitochondrial and six other proteins (CRYAB, DDX3X, HTRA2, PDZK8, ANK2, FEZ1)—occasionally mitochondrial.

### Identification and functional characterization of the HAX1 interactome obtained by affinity purification and mass spectrometry

**Figure 1:**
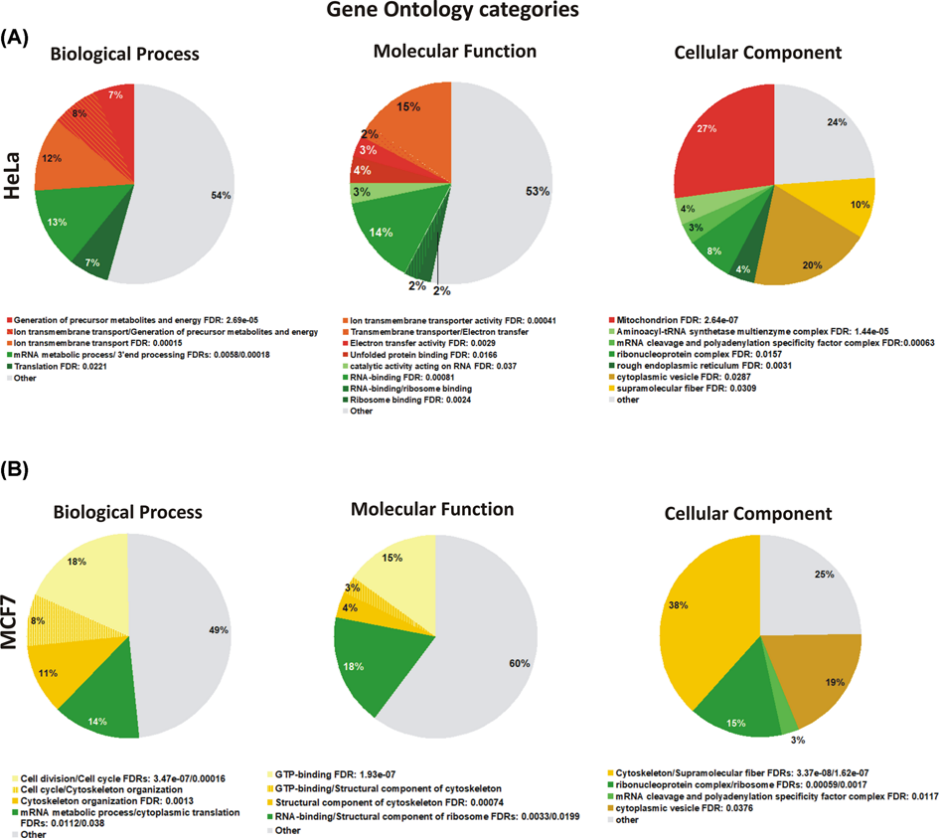
Gene Ontology analysis showing the enrichment in biological process, molecular function and cellular localization Gene Ontology analysis showing the enrichment in biological process, molecular function and cellular localization in HeLa (**A**) and MCF7 (**B**) cells (AP-MS results). Same processes/functions/localizations are marked in the same colors. Enrichment statistics shown as false discovery rate (FDR). For both cell lines, the enrichment was found only in categories involved in RNA binding and RNA processing/translation, the other categories displayed substantial differences. Generated using STRING 11.0.

**Figure 2:**
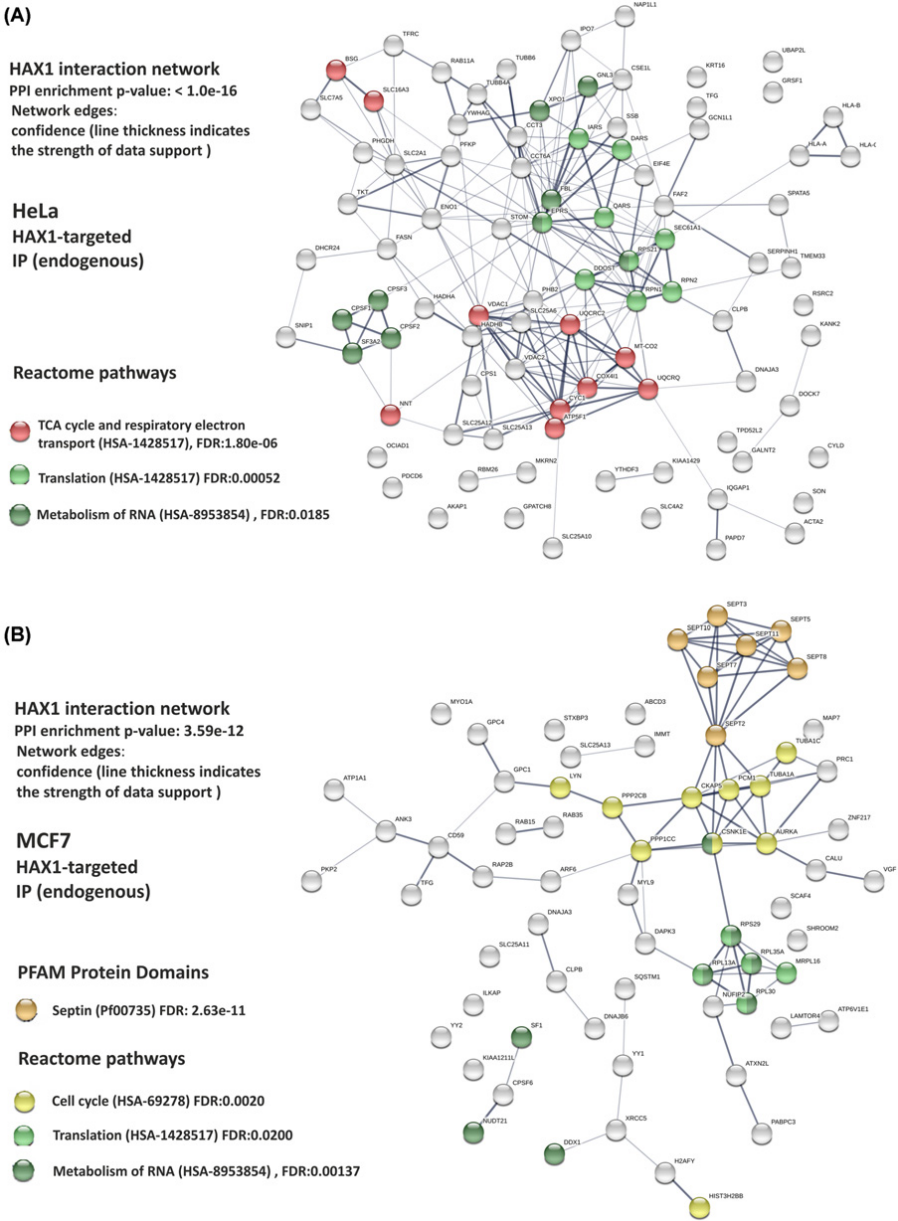
STRING analysis of the HAX1 interaction network in HeLa and MCF7 cells (AP-MS results) Proteins of the specified Reactome pathways forming nodes with the most edges are marked. (**A**) Reactome pathways in HeLa: TCA cycle and respiratory electron transport, translation, RNA metabolism. (**B**) Reactome pathways in MCF7: cell cycle, translation and RNA metabolism. Septin cluster, representing a distinct PFAM protein domain is marked separately, although septins are also involved in the regulation of cell cycle. Generated using STRING 11.0. Abbreviation: TCA cycle, tricarboxylic acid cycle.

As an alternative approach, HAX1-targeted affinity purification and mass spectrometry (AP-MS) analysis was performed independently in the two human tumor cell lines derived from cervical carcinoma (HeLa) and breast cancer (MCF7). In case of HeLa cells, the additional negative control with *HAX1* knockout (*HAX1* KO, Supplementary Figure S3) was available and was used with anti-HAX1 antibody to improve specificity. Three independent repeats of the experiment and of the appropriate controls were analyzed for each cell line. The results were sorted for relevance according to the combined outcome of the Mascot score, number of peptides and the coverage (Supplementary File S4). The resulting datasets of 92 proteins (HeLa) and 73 proteins (MCF7) were analyzed, revealing substantial discrepancies, with only 4 overlapping proteins (CLPB, SLC25A13, DNAJA3, TFG), suggesting that HAX1 protein complexes might be cell line-specific. The results of Gene Ontology and STRING analyses of these datasets are depicted in Figures 1 and 2.

#### HeLa interactome

Functional enrichment of biological processes in HeLa cells (Figure 1A, Supplementary File S2) revealed a significant group of proteins involved in mitochondrial processes (generation of precursor metabolites and energy, mitochondrial transport) with other significant groups involved in RNA processing. Molecular function analysis revealed a large group of proteins involved in RNA binding (18% of all proteins in the dataset), including a subset of proteins involved in ribosome binding (4%). Additionally, an overrepresentation of proteins with transmembrane transporter activity (17%) and electron transfer activity (5%) was detected. Cellular component analysis indicated that the two largest groups were represented by mitochondrial proteins (27%) and cytoplasmic vesicle proteins (20%), followed by ribonucleoproteins (8%). Accordingly, STRING analysis performed for this dataset revealed protein clusters associated with Reactome pathways involved in mitochondrial processes (tricarboxylic acid cycle (TCA cycle) and respiratory electron transport) and RNA-related functions (RNA processing and translation) (Figure 2A). Mitochondrial protein subset and the associated pathways represent the most significant results in HeLa cells.

Additionally, HAX1-targeted immunoprecipitation and subsequent MS was performed in HeLa cells for GFP-tagged, overexpressed HAX1. The resulting dataset was consistently enriched with proteins involved in translation, mRNA metabolism and in mitochondrial transport and energy generation, conforming to the results obtained for immunoprecipitation with endogenous protein (Supplementary Figure S4 and File S2).

#### MCF7 interactome

In the protein subset obtained from MCF7 cells the enriched biological processes include cell cycle, cytoskeletal organization, mRNA decay and translation initiation (Figure 1B, Supplementary File S2). Molecular function analysis revealed again an enrichment in RNA-binding factors (18%), but mitochondrial transmembrane transporters were scarce, while the most numerous in this dataset were proteins involved in GTP binding (18%). Distribution of the proteins revealed by cellular component analysis is partially similar to the distribution in HeLa cells, with the second largest group represented by the proteins of cytoplasmic vesicles (19%) and a relatively large group of ribonucleoproteins (15%). However, mitochondrial proteins represent only 8% in this dataset (not appearing as important in GO analysis) and the most numerous group is represented by cytoskeletal proteins (38%), which include a highly significant cluster of six septins (SEPT 2, 3, 5, 7, 8,11) linked to another cluster of proteins associated with microtubules and cell division, associated with AURKA. STRING analysis of this dataset revealed Reactome pathways pertaining to cell cycle, associated with a strong septin cluster and, as in case of HeLa cells, RNA processing and translation (Figure 2B).

### Verification of selected Y2H and AP-MS results by co-immunoprecipitation

#### Y2H results

**Figure 3:**
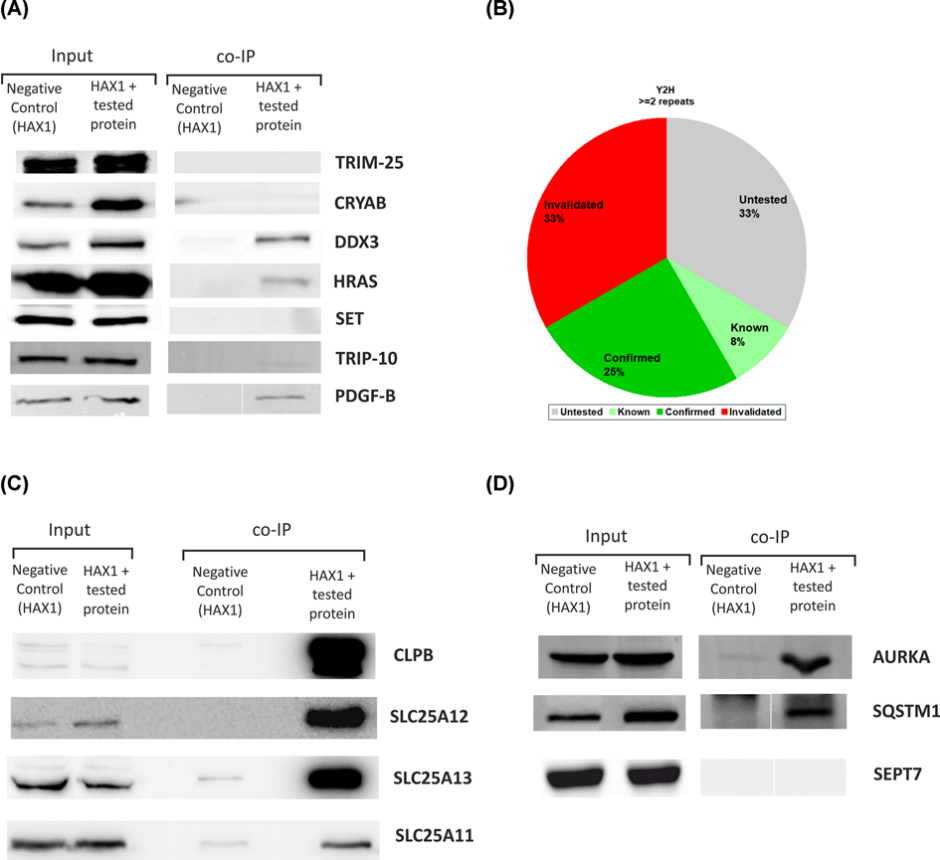
Validation of proteomics data obtained in both screens by co-immunoprecipitation of the tagged HAX1 with the selected, tagged target proteins The layout of the experiments with tags and antibodies used for detection is presented in Supplementary Table S2. (**A,B**) Two-hybrid system results. (A) Western blots confirming three of the seven tested interactions (HRAS, PDGFB, DDX3X). (B) Proportion of HAX1 interacting proteins that were confirmed or invalidated by co-immunoprecipitation experiments with tagged HAX1 in HeLa cells. (**C,D**) Validation of the selected AP-MS results. (C) Mitochondrial subset containing the chaperone (CLPB) and the transporters from malate-aspartate shuttle. (D) Verification of the interactions of AURKA, sequestosome (SQSTM1) and septin 7 (SEPT7).

Y2H results were sorted by relevance, according to the number and variability of repeats and biological significance. Eight proteins, described in Table 1, were chosen for further analysis: CLPB, CRYAB, DDX3X, HRAS, PDGFB, SET, TRIM25, TRIP-10. Chosen hits were cloned and their respective constructs were analyzed by a co-transfection with HAX1-bearing plasmid in HeLa cells and a subsequent co-immunoprecipitation of the tagged constructs. Four interactions were confirmed by immunoprecipitation (CLPB, HRAS, PDGFB and DDX3X, Figure 3A,B).

**Table 1:** Y2H results obtained in two or more repeats

Protein symbol	Protein name	Repeats	Library	Bait/Prey	co-IP
CRYAB	crystallin, α B	11	Hippocampus Hypothalamus	Prey	-
PDGFB	platelet-derived growth factor subunit B	6	Breast/Prostate Cancer	Bait	+
CLPB	ClpB homolog, mitochondrial AAA ATPase chaperonin	5	Brain, Hippocampus	Bait	+
CLOCK	clock circadian regulator	3	Hypothalamus	Bait	
TRIM25	tripartite motif containing 25	3	Breast cancer	Bait	-
TRIP10	thyroid hormone receptor interactor 10	3	Liver	Bait	-
HRAS	Ras oncogene family	2	Breast /Prostate cancer	Bait	+
EPB41L3	erythrocyte membrane protein band 4.1-like 3(1087)	2	Brain	Prey	
DDX3X	DDX3X RNA helicase	2	Liver	Bait	+
BIRC3	baculoviral IAP repeat-containing 3	2	Breast cancer	Bait	
SET	nuclear proto-oncogene	2	Breast cancer	Bait	-
STAM2	signal transducing adaptor molecule 2	2	Spleen	Bait	
ANP32B	acidic (leucine-rich) nuclear phosphoprotein 32 family member B	2	Brain, Hypothalamus	Prey	

#### AP-MS results

From protein hits obtained by mass spectrometry several were selected for further analysis according to their reproducibility, confidence of interaction according to the position on the scoring list (dependent on number of peptides, probability-based Mascot score and coverage) and/or biological significance. Seven proteins were chosen for verification: CLPB, SLC25A13 (from both analyses: HeLa nad MCF7), SLC25A12 (HeLa), SEPT7, AURKA, SQSTM1, SLC25A11 (MCF7 cells). Co-immunoprecipitation with the tagged constructs confirmed six interactions (Figure 3C,D).

### Enrichment in RNA-binding proteins in HAX1 interactome suggests that HAX1 may bind RNA

**Figure 4:**
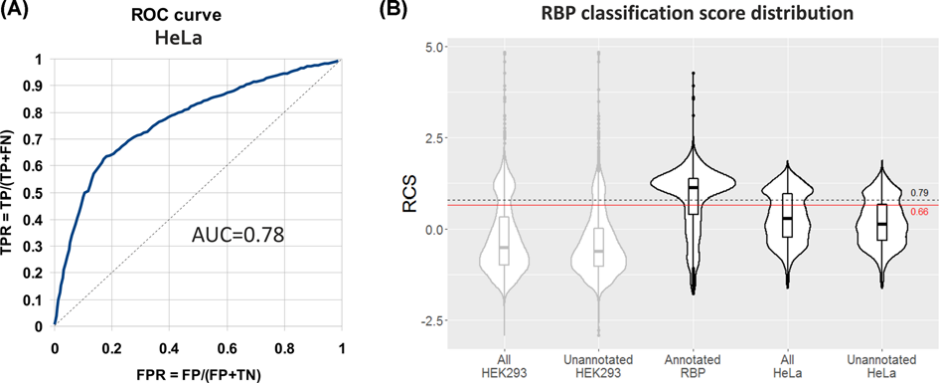
HAX1 classification score obtained by SONAR analysis is on the threshold of the score assigned for RNA-binding proteins (**A**) ROC-AUC analysis of classifier performance for human proteins from HeLa dataset [48]. (**B**) Violin plots of RCS distributions for human proteins analyzed in HEK293 and HeLa datasets. All proteins and unannotated (non-RNA-binding, true negatives) for both datasets are plotted along with the group of annotated proteins (true positives). Optimal cutoff for HeLa dataset, for both specificity (0.74) and sensitivity (0.69) was established using ROC curve as a value of 0.66 RCS (±0.3), which puts a value for HAX1 (0.63 RCS) within the margins of this threshold.

AP-MS results obtained for both cell lines indicate a high proportion of RNA-binding proteins and the potential involvement in RNA processing and translation. Support-vector-machine Obtained from Neighborhood Associated RBPs (SONAR) was proposed by Brannan et al. [44] to calculate RCS, which corresponds to the RNA-binding potential of the protein and is determined by the analysis of the large scale PPI networks. SONAR analysis was performed using the parameters and the datasets from the original study (HEK293, [47]) and from the study by Hein et al. (HeLa, [48]), both modified to include HAX1 interactions obtained for HeLa cells in the current study. RCS calculated for HAX1 with the proteomic results obtained in the present study demonstrated an increase from 0.29 for original HEK293 dataset and 0.21 for HeLa dataset to 1.09 and 0.63, respectively. The RCS threshold proposed by Brannan et al. [44] for HEK293 dataset was set at 10% false positive ratio (FPR) at the RCS value of 0.79. While this threshold was appropriate for the global study where specificity was of utmost importance; in our study, in which we are trying to assess the probability of RNA-binding for a specific protein, sensitivity also should be considered. Assigning 10% FPR for the HeLa dataset results in high specificity, but no sensitivity (0.9 and 0.5, respectively). Thus, to establish a new cutoff, we have calculated optimal specificity/sensitivity cut-off point from the ROC curve (Figure 4A) of the HeLa dataset, obtaining the RCS value of 0.66 (specificity 0.74, sensitivity 0.69). HAX1 RCS value calculated in HeLa background amounts to 0.63, so it lies within the margins of the calculated threshold (Figure 4B). In both cases, for HeLa and HEK293 datasets the RCS for HAX1 is significantly higher for our dataset than for the pre-existing one. Two other predictions performed to assess RNA-binding properties of HAX1: RNApred (based on amino acid sequence) and catRAPID (based on structural, physical and chemical properties) resulted in classification of HAX1 as RBP (Supplementary Figure S5). CatRapid prediction also points to a region with a potential β strands (194–259 aa) as a possible RNA-binding site (Supplementary Figure S5).

### Mitochondrial subset of HAX1-interacting proteins suggests HAX1 involvement in metabolism and protein stabilization

#### Mitochondrial subset characteristics

Mitochondrial proteins are present mostly in the AP-MS results from HeLa cells, where they constitute ∼27% of positive hits. Mitochondrial proteins obtained in the experiment are involved in energy and metabolites generation and aggregation control. AP-MS results from HeLa cells represent the only dataset obtained so far in HAX1 interactome studies significantly enriched in mitochondrial proteins. This mitochondrial subset includes transmembrane transporters forming malate-aspartate shuttle (MAS) pathway, proteins of electron transport chain (ETC) and mitochondrial chaperones. MAS proteins (SLC25A11,12,13 and associated SLC25A10) were never previously reported as a part of HAX1-interactome, but represent high-scoring hits, present in both cell lines and were confirmed by co-immunoprecipitation (Figure 3C). Another set of metabolically active proteins detected in the analysis is represented by ETC proteins (UQCRC2, UQCRQ, CYC1, COX4I1, MT-CO2, NNT), although they all have relatively low Mascot score (56∼287). Mitochondrial subset from HeLa cells (21 proteins) was analyzed by Enrichr GO Biological Process, showing the enrichment not only in amino acid transport, ETC and oxidative phosphorylation, but also in cardiolipin metabolism, arginine and citrulline biosynthesis (urea cycle) and gluconeogenesis (Supplementary File S2).

The th3ird important group of mitochondrial proteins detected in the analysis comprises chaperones. Mitochondrial chaperones obtained in this analysis (CLPB and DNAJA3) were detected in both cell lines (CLPB was also detected in Y2H analysis). Additionally, in AP-MS from HeLa, a previously described interaction with a chaperone prohibitin (PHB2) [25] was confirmed.

#### Interaction with CLPB

**Figure 5:**
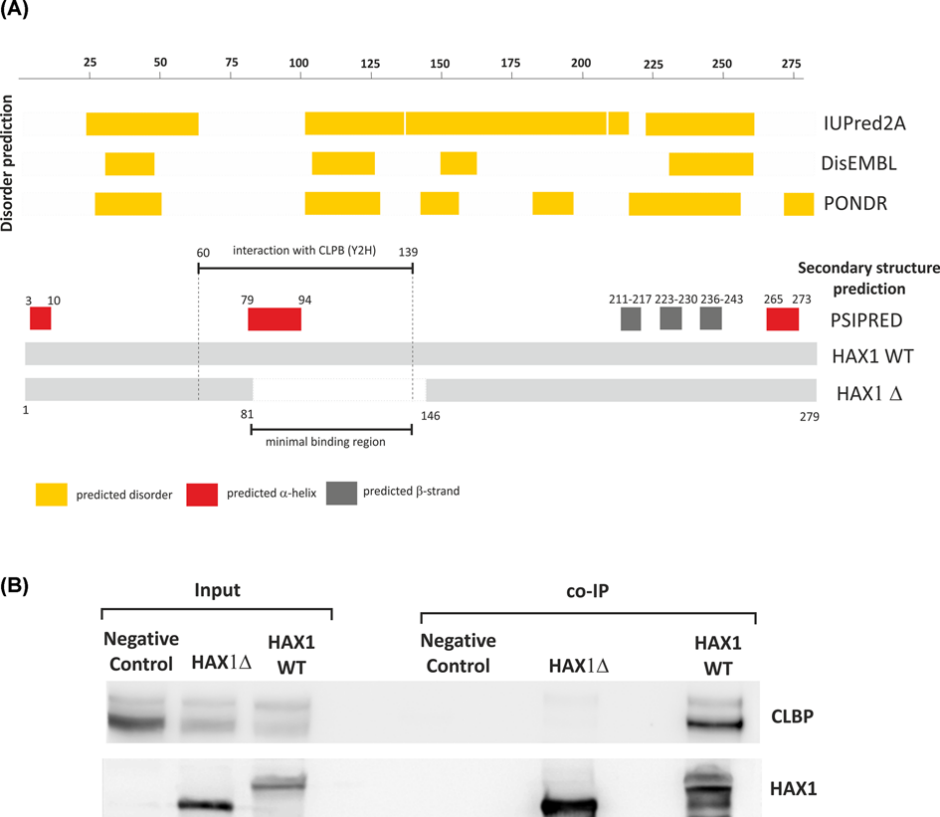
Mapping of the HAX1–CLPB interaction (**A**) Prediction of secondary structure elements and structural disorder in HAX1 in relation to CLPB binding region. The region predicted as interacting with CLPB according to Y2H, the region of the deletion which precludes CLPB binding (81–146 aa) and the minimal binding region are marked. (**B**) Co-immunoprecipitation with the whole HAX1 and the 81–146 aa deletion, showing minimal interaction for the deletion.

CLPB represents the strongest result, present in all analyses. The interaction with CLPB was further analyzed, since it was the only protein for which the potential interacting region was mapped in Y2H to the middle part of HAX1 (Figure 5A). Secondary structure analysis predicted that this region (60–139 aa) contains the longest structural motif present in HAX1—an α-helix in position 79–94 aa. Besides this potential structural motif, the rest of this region is predicted to be disordered (Figure 5A). The co-immunoprecipitation of HAX1 with the deletion of 81–146 aa, containing the predicted binding region with the α-helix, demonstrated that the deletion precludes CLBP binding (Figure 5B). This suggests that the minimal CLPB-binding region in HAX1 can be preliminarily mapped to 81–139 aa.

### HAX1 has a role in cellular response to oxidative stress and aggregation control

#### Oxidative stress

**Figure 6:**
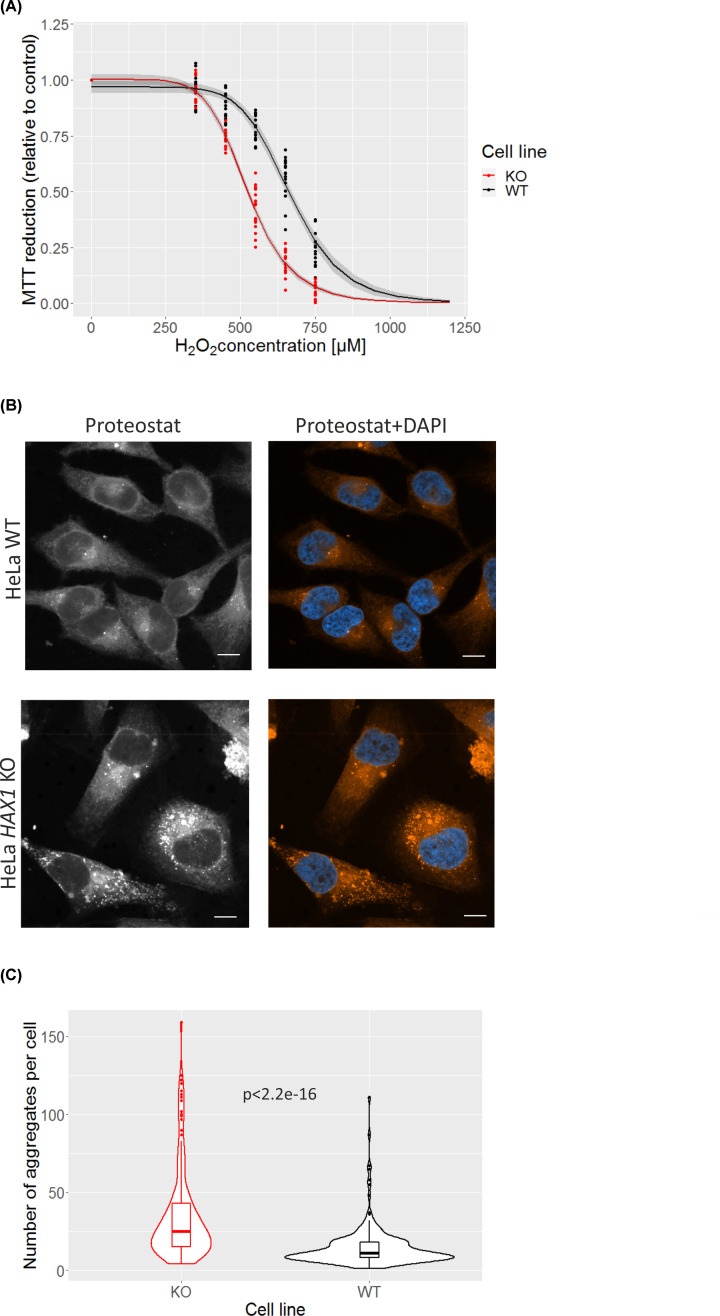
*HAX1* knockout affects a response to oxidative stress and protein aggregation (**A**) Dose–response curve for HeLa WT and HeLa *HAX1* KO cells treated with increasing H_2_O_2_ concentrations. Dots on the graph represent normalized results obtained from four independent MTT assays. Gray ribbons represent 95% confidence intervals. Significance assessed with Welch’s two sample *t*-test for each H_2_O_2_ concentration (350, 450, 550, 650, 750, respective *P*-values: 0.5021, 4.75E-09, 1.33E-12, 9.20E-13, 1.43E-08). (**B**) *In situ* protein aggregates detection. Aggregates stained with Proteostat, nuclei stained with Hoechst 33342. Scale bar: 10 μm. (**C**) Violin and box plots representing quantification of intracellular protein aggregates detected in HeLa WT and HeLa *HAX1* KO cells. For each cell line protein aggregates from ≈200 cells from three independent experiments were quantified. Difference between cell lines was assessed by Mann–Whitney U test (*P*-value <2.2e-16).

The presence of the prominent mitochondrial subset in the HAX1 interactome, suggests that HAX1 may have a role in the regulation of mitochondrial stability and metabolic functions. An MTT assay performed for HAX1 WT and *HAX1* KO HeLa cells subjected to oxidative stress by increasing concentration of H_2_O_2_, demonstrated significant differences in the susceptibility to the oxidative stress, with the decreased viability of the knockout cells (Figure 6A).

#### Protein aggregation

The strongest HAX1 interaction characterized here is the chaperone CLPB (de-aggregase), and the HeLa dataset contains overall seven proteins of chaperone activity (7.6%) including three mitochondrial chaperones. These results suggest that HAX1 may be involved in the control of protein aggregation. Protein aggregates were detected by Proteostat fluorescent dye and the number of aggregates were compared between HeLa WT and *HAX1* KO, showing increased aggregation in *HAX1* KO (Figure 6B,C). Moreover, a rescue experiment with WT HAX1 (vs. control plasmid) performed in HAX1 KO background resulted in aggregation status shifted toward decreased number of aggregates (Supplementary Figure S6).

## Discussion

HAX1 is a multifunctional protein involved in many cellular processes [1] but the molecular mechanism behind this involvement still awaits explanation. The problem with pinpointing HAX1 molecular function may stem from the fact that it is intrinsically disordered [34,35]. Intrinsically disordered proteins (IDPs) usually display multiple, but relatively weak interactions. So far, 40 HAX1 interactions have been confirmed in the literature and databases report ∼200 potential interactions; such large number of potential interactions conforms to the HAX1 IDP profile.

**Figure 7:**
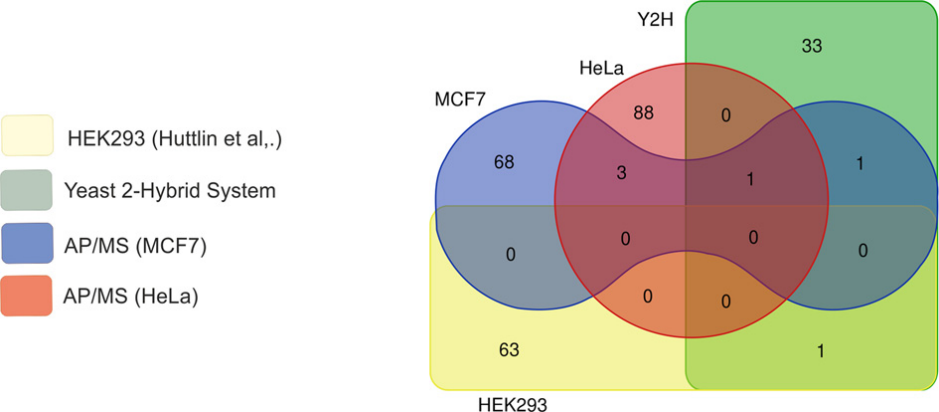
HAX1 interactomes from different cell types display high variability Venn diagram showing the number of unique and shared proteins indicates low reproducibility of the identified interactions between different cell types. Compared datasets: HEK293 (Huttlin et al. [47]), results from this study: HeLa, MCF7, Y2H (libraries from different tumors).

In the current report, we characterized overall 197 potential interactions of HAX1 and confirmed 9. We have analyzed and compared the results of the Y2H screen performed for several human libraries derived from different tissues (mostly the brain) with AP-MS results from the two human cell lines (breast and cervical cancer). The results show low reproducibility, with only one protein (CLBP) present in all analyses. We have compared our analysis with the data from the BioGRID database, the bulk of which was obtained by Huttlin et al. [47] for HEK293 cell line, derived from human embryonic kidney cells with certain neuronal characteristics [49]. These results indicate very specific enrichment in proteins involved in neuronal development, synaptic vesicles and vesicular transport. The only common feature of this dataset and the results presented here is a representation of proteins involved in vesicular transport. Since the overlap observed for all studied datasets is small, and the datasets were obtained for different cell types, we propose that HAX1 interactomes may differ depending on a studied tissue and include many proteins which display relatively weak binding, which is characteristic to IDPs (Figure 7).

Enrichment analyses performed on the datasets obtained in AP-MS experiments indicate that despite low reproducibility of the specific results, two categories of proteins persist in both analyzed cell lines: proteins involved in translation and RNA processing (Figure 7B,D). This result may be linked to RNA-binding properties of HAX1 reported previously [29,31]. In this study, the probability of RNA-binding by HAX1 was tested using a proteomic approach based on the analysis of the interactome of the given protein in the context of the whole PPI network. The results showed a significant increase in the RCS for the proposed HAX1 interactome in HeLa cells, placing it on the threshold for the RNA-binding proteins. The other two prediction analyses, based on different algorithms, classified HAX1 as RBP. These results, while not very definite, suggest that HAX1 binding to other RBPs may indicate that the protein is an RBP itself, as suggested by Brannan et al. [44]. However, low reproducibility suggests that these interactions are likely to be weak and occur in a tissue-specific manner. Since HAX1 does not possess any known RNA-binding domain it may be proposed that RNA binding occurs via a region with the three predicted β strands but also with a relatively high disorder (CatRapid prediction, Supplementary Figure S5B). As suggested by Gene Ontology analyses, HAX1 binding to other RBPs and RNA may indicate its involvement in the regulation of RNA metabolism.

Except for translation and RNA processing our analyses detected a group of cytoskeletal proteins involved in cell cycle regulation in MCF7 cells and a mitochondrial subset in HeLa cells, with the minimal overlap between these groups, which point again to cell line-specific interactions (Figure 7C,E). Notably, for HeLa cells, AP-MS experiments performed for endogenous and GFP-tagged protein consistently show the enrichment (or the lack of thereof) in the same protein pathways (Supplementary Figure S7). Hitherto, HAX1 was reported to interact with cytoskeletal components [13] and has been implicated in actin rearrangement [19]. In MCF7 cells, HAX1 is predicted to interact with 18 cytoskeletal proteins, mostly implicated in cell cycle, including 7 septins and 5 proteins which constitute Aurora A activation cluster. In our co-immunoprecipitation experiment we did not confirm the interaction with septin 7, but it might be an effect of the heteromeric structure of septin filaments; in our analysis we have found 7 septins in the endogenous settings, so it seems possible that the specific overexpression of a single septin in the confirmatory experiment creates a stoichiometric imbalance in septin composition, precluding interaction. In our earlier study we have shown that HAX1 and septin 7 co-localize in MCF7 cells and that HAX1 deficiency affects cellular distribution of septin 7 and septin 2, supporting the notion of their connection [14]. Large cytoskeletal subset is also present in HAX1 interactome obtained for HEK293, but there is no overlap with MCF7 and biological process analysis reveals that proteins from HEK293 dataset are involved mostly in neuron development, synaptic vesicles and neurotransmitter secretion, and not the cell cycle, which underlines the conclusion of the plasticity and tissue-specificity of HAX1 interactomes.

HAX1 has been observed in different cellular localizations, but there is a consensus that it is predominantly mitochondrial [25,26]. Thus, the data provided hitherto by the literature and high-throughput analyses on HAX1 interactome seem incomplete, since they do not contain any substantial group of mitochondrial proteins. In the analyzes presented here we provide for the first time a mitochondrial subset of the 21 proteins (out of 92) potentially interacting with HAX1, isolated from HeLa cells. Concurrent analysis in MCF7 cell line confirmed several of these interactions (CLPB, SLC25A13, DNAJA3, interaction with a similar protein SLC25A11), but the overall number of mitochondrial hits was significantly lower (6 out of 73). One of the main findings presented here is the characterization of this mitochondrial subset of the potential HAX1-interacting proteins, with a three distinct, but possibly cooperating groups; associated with the MAS, the ETC and the control of aggregation (chaperones). The MAS translocates electrons produced during glycolysis across the inner mitochondrial membrane and its malfunction can affect oxidative phosphorylation. It is also associated with the urea cycle, since the aspartate provided by this shuttle is an immediate substrate for argininosuccinate synthetase (ASS1), a urea cycle enzyme. Additionally, mitochondrial protein carbamoyl phosphate synthetase (CPS1), present in the obtained HeLa interactome, represents an enzyme catalyzing the first step in the urea cycle. It is possible that MAS and ETC proteins cooperate in the regulation of mitochondrial metabolism and, possibly, the metabolic shift between glycolysis and oxidative phosphorylation and that HAX1 plays a role in this regulation. Cell viability assay confirmed that *HAX1* KO is more susceptible to oxidative stress than unmodified cell line (Figure 6A), but further research is needed to clarify what pathway is responsible for this effect.

The third group of mitochondrial proteins detected in the present study comprise mitochondrial chaperones (CLPB, PHB2, DNAJA3), with the strongest hit, CLPB, and prohibitin (PHB2), both of which have been already reported as binding to HAX1 [25,50]. Previous reports also indicate that HAX1 interacts with other proteins responsible for mitostasis and control over mitochondrial unfolded protein response (mtUPR) and aggregation: HSPA9 [51], HSP90AB1 [52] and HTRA2 [11]. CLPB was identified in all experiments presented here, including the yeast two-hybrid system. The analysis of the length and position of the positive clones obtained in the two-hybrid system enabled preliminary mapping of the CLPB-binding region within HAX1, which was subsequently narrowed to a region containing the longest predicted α-helix. This is unique for HAX1, because most of the mapped so far regions of interaction are in the disordered, C-terminal part of the protein [1]. Thus, we propose, that the interaction with CLPB is exceptional for HAX1, because it is very strong and mediated by the most pronounced structural element of this protein, in contrast with the many weak interactions mapped to the disordered part.

Strong interaction suggests that HAX1 may affect CLPB functions in the mitochondria. CLPB promotes protein de-aggregation [53]. Cupo and Shorter [54] suggested recently that CLPB couples ATP hydrolysis to protein disaggregation and reactivation and that its deficiency affects HAX1 solubility. Additionally, another HAX1-binding protein HSPA9 (mtHSP70, [51]) prevents aggregation of proteins. Moreover, HAX1-binding PHB2 was recently identified as a novel mitophagy receptor that is affected by mitochondrial membrane depolarization or misfolded protein aggregation [55]. Together these results indicate that HAX1 may be involved in the regulation of protein aggregation. Accordingly, preliminary results presented here demonstrated enhanced aggregation in *HAX1* KO cells, compared with unmodified HeLa cells (Figure 6B,C, Supplementary Figure S6).

In conclusion, presented analyses indicate that HAX1 binds proteins in a tissue-specific manner and its interactomes in MCF7 and HeLa cells may include proteins involved in RNA metabolism (both cell lines), cytoskeleton regulation (MCF7) and energy generation in the mitochondria (HeLa). A subset of HAX1 protein partners comprises chaperones, suggesting its involvement in protein aggregation. These results conform to HAX1 disordered characteristics, suggesting a wide spectrum of weakly bound protein targets and a high plasticity of protein complexes formed in different tissues. Moreover, in the present study we present for the first time the mitochondrial subset of HAX1 interactome.

## Supplementary Material

Supplementary Tables S1-S2 and Supplementary Figures S1-S7

Supplementary Files S1-S4

## Abbreviations

AP-MS: affinity purification and mass spectrometry
AUC: area under curve
AURKA: Aurora A kinase
BioGRID: Biological General Repository for Interaction Datasets
CLPB: caseinolytic peptidase B protein homolog
ETC: electron transport chain
FPR: false positive ratio
HAX1: HCLS1-associated protein X-1
IDP: intrinsically disordered protein
KO: knockout
MAS: malate-aspartate shuttle
PPI: protein–protein interaction
PSM: peptide spectrum match
RBP: RNA-binding Protein
RCS: RBP classification score
ROC: receiver operating characteristic
SONAR: Support-vector-machine Obtained from Neighborhood Associated RBP
STRING: Search Tool for the Retrieval of Interacting Genes/Protein
Y2H: yeast two-hybrid screen
