# Supplementary material for: The interactome of multifunctional HAX1 protein suggests its role in the regulation of energy metabolism, de-aggregation, cytoskeleton organization and RNA-processing
Source: Biosci Rep. 2020 Nov 13;40(11):BSR20203094. doi: 10.1042/BSR20203094 (PMC7670567; doi:10.1042/BSR20203094)
Supplement: Supplementary Tables S1-S2 and Supplementary Figures S1-S7 [file BSR-2020-3094_supp.pdf]

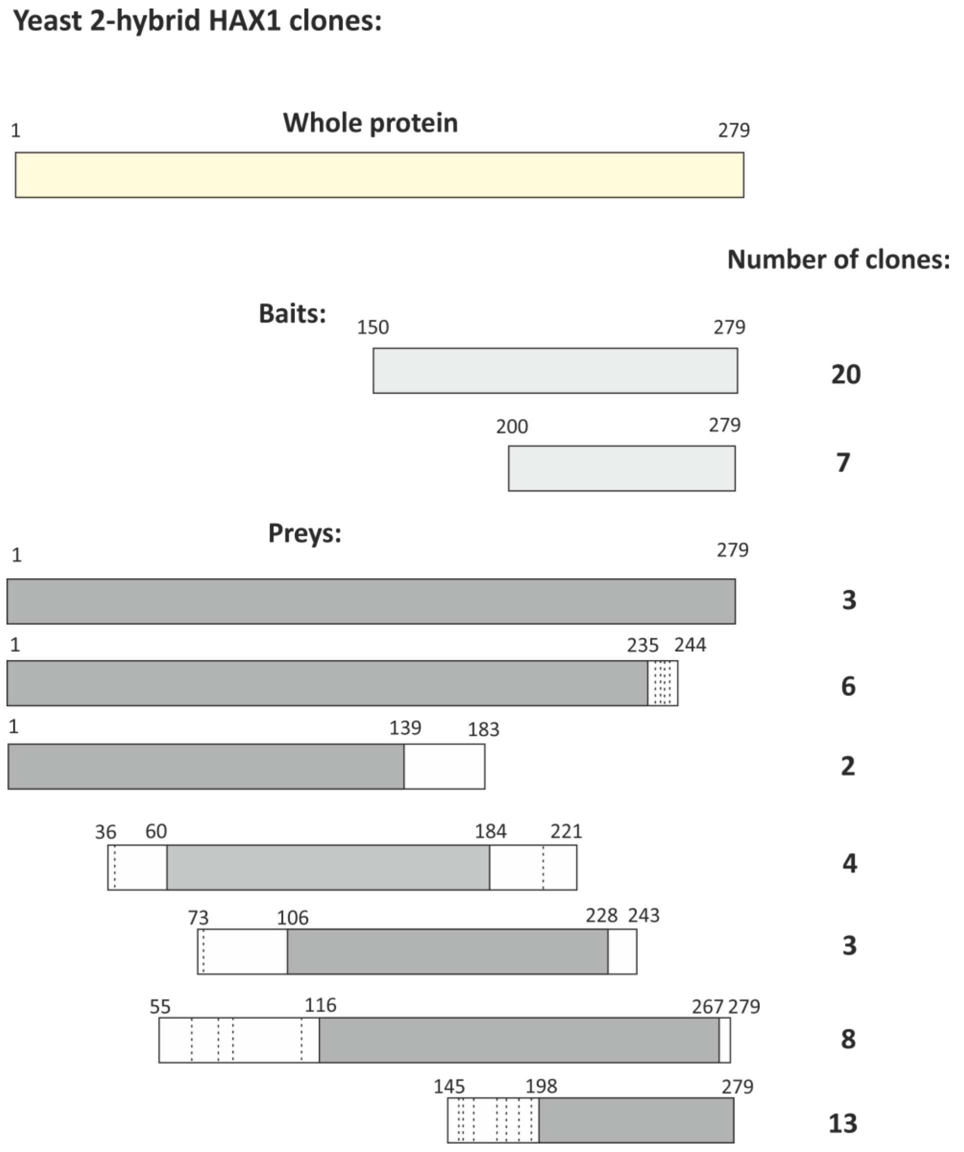

Figure S1. Representation of the Y2H HAX1 clones (baits and preys) in comparison to the whole protein (yellow). Baits are arbitrary designed at the C-terminus, most of prays also encompass C-terminal part. 5 of the 6 clones from N-terminus and the middle part interact with CLPB.

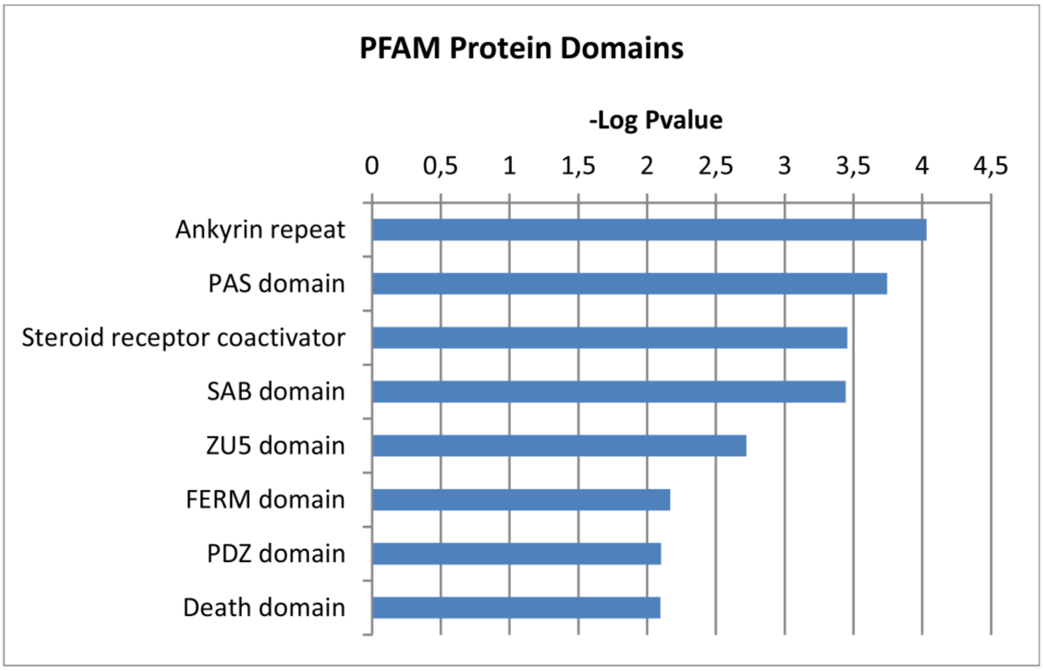

Figure S2. PFAM protein domains significantly enriched within HAX1 binding partners obtained by Y2H. Domains were identified using STRING web resource. The most significant group is represented by proteins with ankyrin repeats (FDR 9.33E-05), a common protein-protein interaction motifs. The other groups include PAS domain (molecular sensor), SAB and FERM domains (cytoskeletal-associated), ZU5 domain (associated with death domain and ankyrins), PDZ domain and death domain.

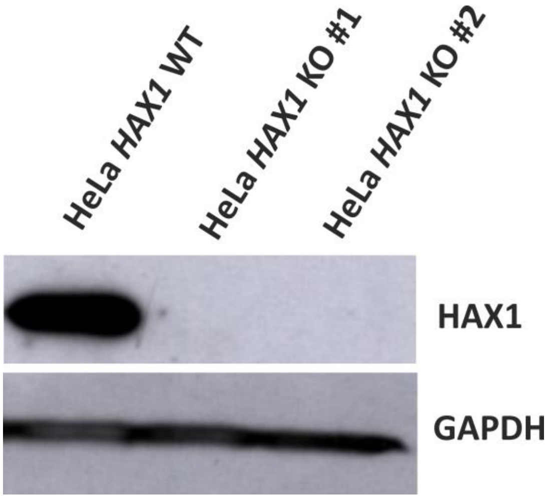

Figure S3. ***HAX1*** **knockout in HeLa cells.** Western blot of the two tested*HAX1* KO cell lines, *HAX1* KO #1 was used in the experiments. Reference: GAPDH.

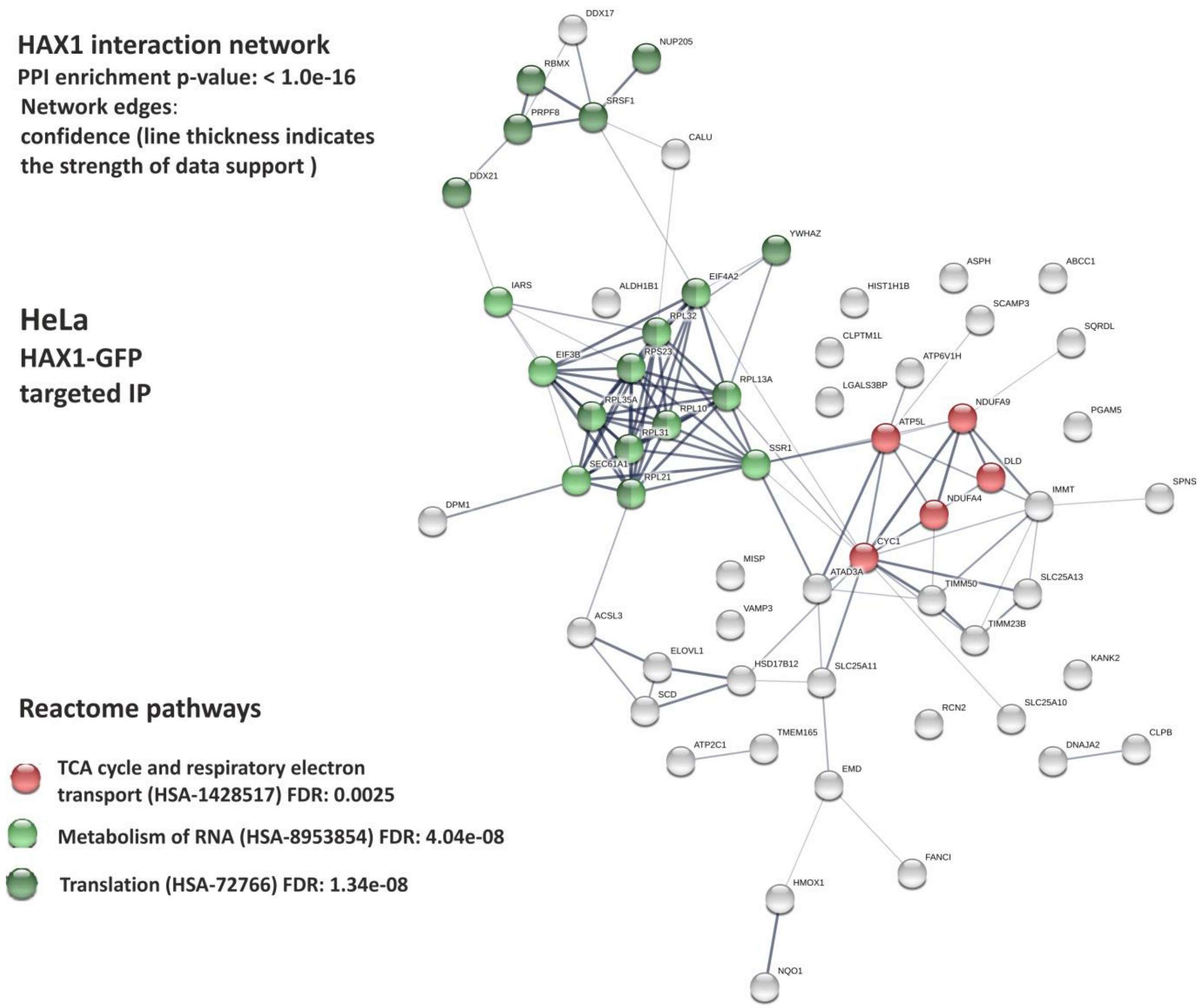

Figure S4. STRING analysis of the HAX1-GFP interaction network in HeLa cells. Proteins of the specified Reactome pathwayspathways (TCA cycle and respiratory electron transport, translation, RNA metabolism), forming nodes with the most number of edges are marked.

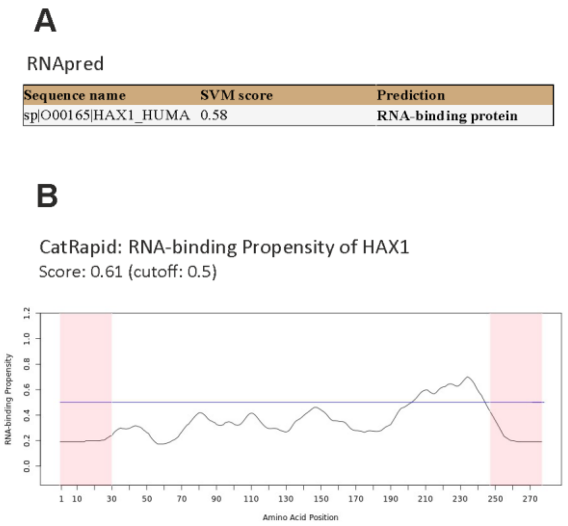

Figure S5. HAX1 is predicted to be an RNA-binding protein by the two independent prediction algorithms. A. RNApred: amino acid composition based SVM prediction for HAX1 B. catRapid: structural-based prediction, identifies a potential RNA-binding region within HAX1 C-terminal part (194-259 aa).

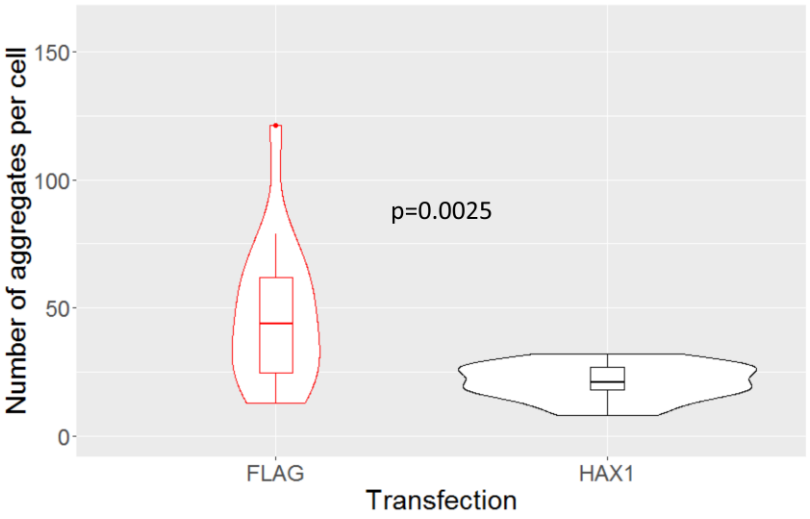

Figure S6. Quantification of intracellular protein aggregates in WT HAX1 rescue experiment. Aggregates were counted inHela *HAX1* KO cells transiently transfected with WT HAX1-FLAG vector (rescue experiment) and the control FLAG vector. For each cell line protein aggregates from ≈ 14-16 cells were quantified. Difference between cell lines was assessed by Mann Whitney U test (p-value=0.0025).

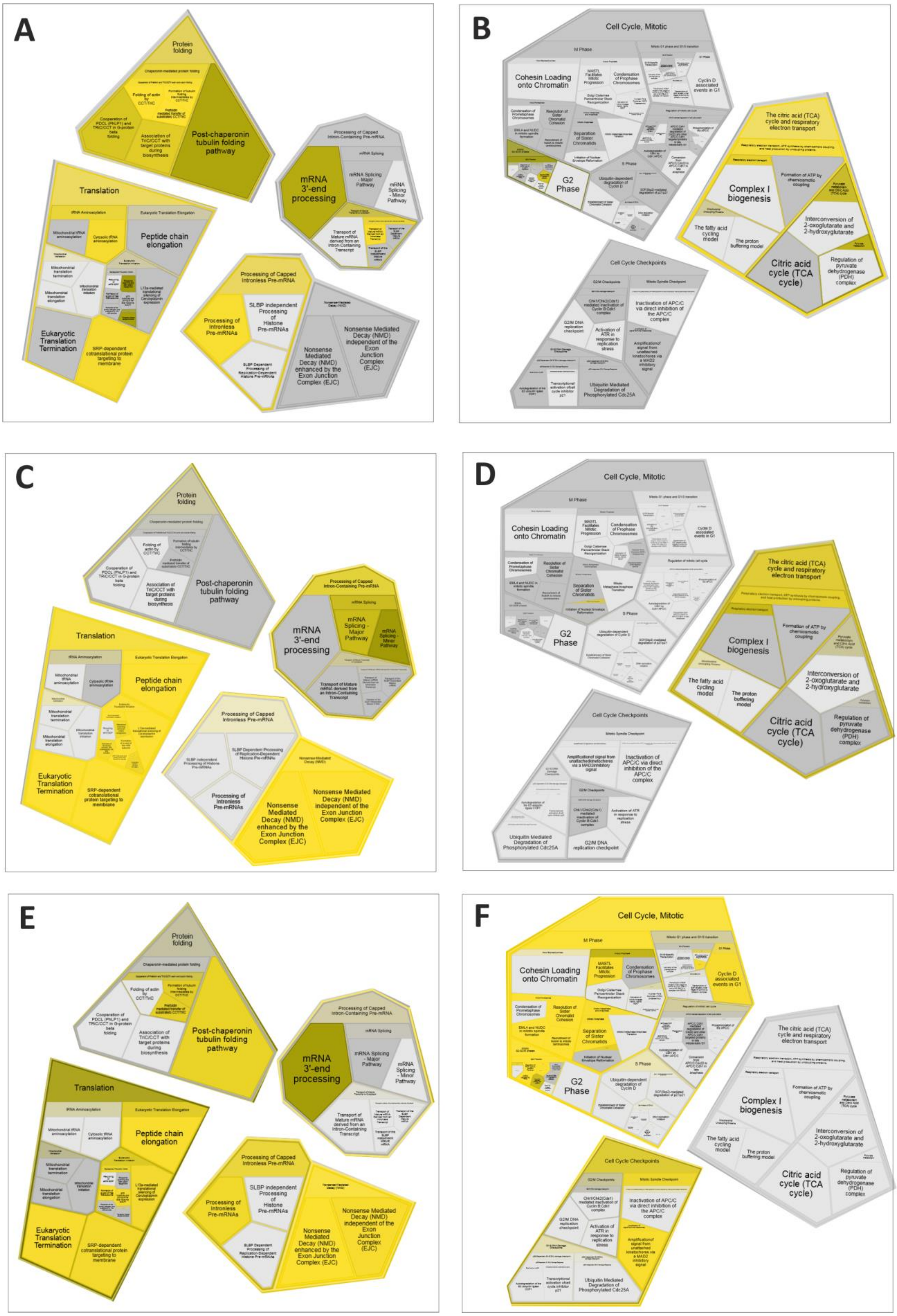

A
Protein folding
Folding of actin by CCT/TriC
Cooperation of PDCL (PhLP1) and TRiC/CCT in G-protein beta folding
Association of TriC/CCT with target proteins during biosynthesis
Post-chaperonin tubulin folding pathway
Translation
Peptide chain elongation
Eukaryotic Translation Termination
SRP-dependent cotranslational protein targeting to membrane
Processing of Capped Intron-Containing Pre-mRNA
mRNA 3'-end processing
mRNA Splicing - Major Pathway
mRNA Splicing - Minor Pathway
Transport of Mature mRNA derived from an Intron-Containing Transcript
Processing of Capped Intronless Pre-mRNA
Processing of Intronless Pre-mRNAs
SLBP independent Processing of Histone Pre-mRNAs
Nonsense Mediated Decay (NMD) enhanced by the Exon Junction Complex (EJC)
Nonsense Mediated Decay (NMD) independent of the Exon Junction Complex (EJC)
B
Cell Cycle, Mitotic
M Phase
Cohesin Loading onto Chromatin
MASTL Facilitates Mitotic Progression
Condensation of Prophase Chromosomes
Cyclin D associated events in G1
Separation of Sister Chromatids
G2 Phase
Ubiquitin-dependent degradation of Cyclin D
S Phase
The citric acid (TCA) cycle and respiratory electron transport
Complex I biogenesis
Formation of ATP by chemiosmotic coupling
Interconversion of 2-oxoglutarate and 2-hydroxyglutarate
The fatty acid cycling model
The proton buffering model
Citric acid cycle (TCA cycle)
Regulation of pyruvate dehydrogenase (PDH) complex
Cell Cycle Checkpoints
G2/M Checkpoints
Mitotic Spindle Checkpoint
Inactivation of APC/C via direct inhibition of the APC/C complex
G2/M DNA replication checkpoint
Activation of ATR in response to replication stress
Ubiquitin Mediated Degradation of Phosphorylated Cdc25A
C
D
E
F

Figure S7. HAX1 interactomes display specific pathway enrichments depending on the studied cell line. Reactome (Voronoi Tree-maps) representation of the enrichment in the specific pathways in HeLa and MCF7 cells. A,B – HeLa (endogenous HAX1), C,D- Hela (HAX1-GFP), E,F – MCF7. While some pathways are consistently enriched (translation, protein folding, pre-mRNA processing) for both cell lines (A,C –HeLa, E- MCF7), some are cell-line specific (TCA cycle and respiratory electron transport in HeLa - B,D cell cycle in MCF7 - F).

Gene	Tag	Backbone	Restriction sites:	Primer Forward (5'-3'):	Primer Reverse (5'-3'):
CLPB	FLAG	pcDNA FL	Bgl II, XhoI	GCGTCCAAGATCTGCCTCGAAGCAGCTGTCAAGATG	GAGCTCCGGTACCTCGAGGATGGTGTTGCACACCTTCTC
CRYAB	FLAG	pcDNA FL	XhoI, EcoRI	GACGAATTCATGGACATCGCCATCCACCA	TTACTCGAGCTATTTCTTGGGGCTGCGG
SLC25A13	FLAG	pcDNA FL	BamHI, XbaI	GAATCCAGGATCCATCAACCGGGGCGAATCATGGC	GCAGTACTCTAGACGGAATTCTGTATGGGCCTCCACCAATAGC
SLC25A12	FLAG	pcDNA FL	HindIII, Sal I	GCGTCAGAAGCTTGCATGGCGGTCAAGGTGCAGACAAC	GACTCCGGTACCACGTCGACCTGAGTGGCTGCCACTGCTGCCTTTG
SLC25A11	FLAG	pcDNA FL	Bgl II, Sal I	GCGTCCAAGATCTCGATGGCGGCGACGGCGAGTGC	GAGCTCCGGTACCACGTCGACACAGCCACTGAGGAAGAGAC
TRIP10	FLAG	pCR3.1	Bgl II, Sal I	AGATCTATGGATTGGGGCACTGAGCTG	GTCGACCCAGAAGCAGCAGCCGACAG
PDGFB	FLAG	pCDNA3.1	BamHI, XhoI	GTGGGATCCATGTCGGCATGAATCGCTGCTG	ATGCTCGAGAGGCTCCAAGGGTCTCCTTC
HRAS	FLAG	pCDNA3.1	BglII,XhoI	GCTGATTCAGATCTGGCCCGGAGGAGCGATGACGGAATATAAG	GCGCTCGAGGGAGAGCACACACTTGCAGCTCAT
SET	FLAG	pcDNA FL	HindIII, BamHI	GCAAGCTTCCATGTCGGCGCCGGCGGCCAA	ATGGATCCTTAGTCATCTTCTCCTTCATCC
SEPT7	FLAG	PCR3.1	BamHI, XbaI	TCGGATCCATGTCGGTCAGTGCGAGATCCGCTG	CTCTAGATTTAAAAGATCTTCCCTTTCTTC

Gene	Tag	HAX1-tag	Ab (IP)	Ab (detection)
AURKA	-	FLAG	Ms α-Flag	Rb α-AURKA
CLPB(1)	FLAG	MYC	Ms α-MYC	Ms α-FLAG (HRP)
CRYAB	FLAG	MYC	Ms α-MYC	Ms α-FLAG (HRP)
DDX3	FLAG	GFP	V_HH α-GFP	Ms α-FLAG (HRP)
HRAS	FLAG	GFP	V_HH α-GFP	Rb α-RAS
PDGFB	FLAG	MYC	Ms α-MYC	Ms α-FLAG (HRP)
SET	FLAG	GFP	V_HH α-GFP	Ms α-FLAG (HRP)
SEPT7	FLAG	GFP	V_HH α-GFP	Ms α-FLAG (HRP)
SLC25A11	FLAG	MYC	Ms α-MYC	Ms α-FLAG (HRP)
SLC25A12	FLAG	MYC	Ms α-MYC	Ms α-FLAG (HRP)
SLC25A13	FLAG	MYC	Ms α-MYC	Ms α-FLAG (HRP)
SQSTM1	GFP	FLAG	Ms α-FLAG	MS α-GFP
TRIP-10	FLAG	GFP	V_HH α-GFP	Ms α-FLAG (HRP)
TRIM25	GFP	MYC	V_HH α-GFP	Rb α-HAX1
CLPB(2)	FLAG	GFP	V_HH α-GFP	Ms α-FLAG (HRP)
