# Supplementary material for: The interactome of multifunctional HAX1 protein suggests its role in the regulation of energy metabolism, de-aggregation, cytoskeleton organization and RNA-processing
Source: Biosci Rep. 2020 Nov 13;40(11):BSR20203094. doi: 10.1042/BSR20203094 (PMC7670567; doi:10.1042/BSR20203094)
Supplement: Supplementary Files S1-S4 [file BSR-2020-3094_supp1.zip › BSR-2020-3094_suppSF1.docx]

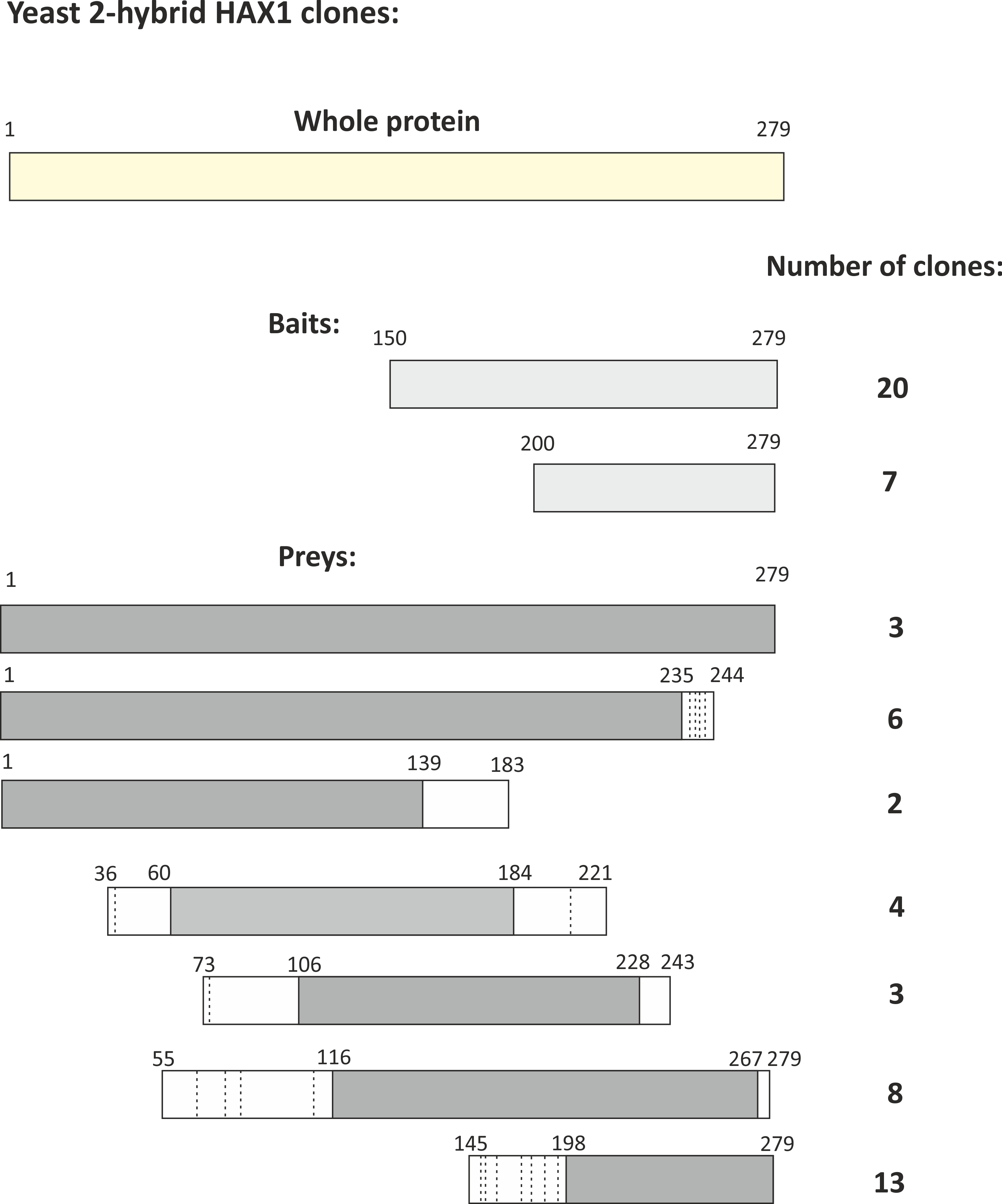


**Figure S1. Representation of the Y2H HAX1 clones (baits and preys) in comparison to the whole protein (yellow).** Baits are arbitrary designed at the C-terminus, most of prays also encompass C-terminal part. 5 of the 6 clones from N-terminus and the middle part interact with CLPB.


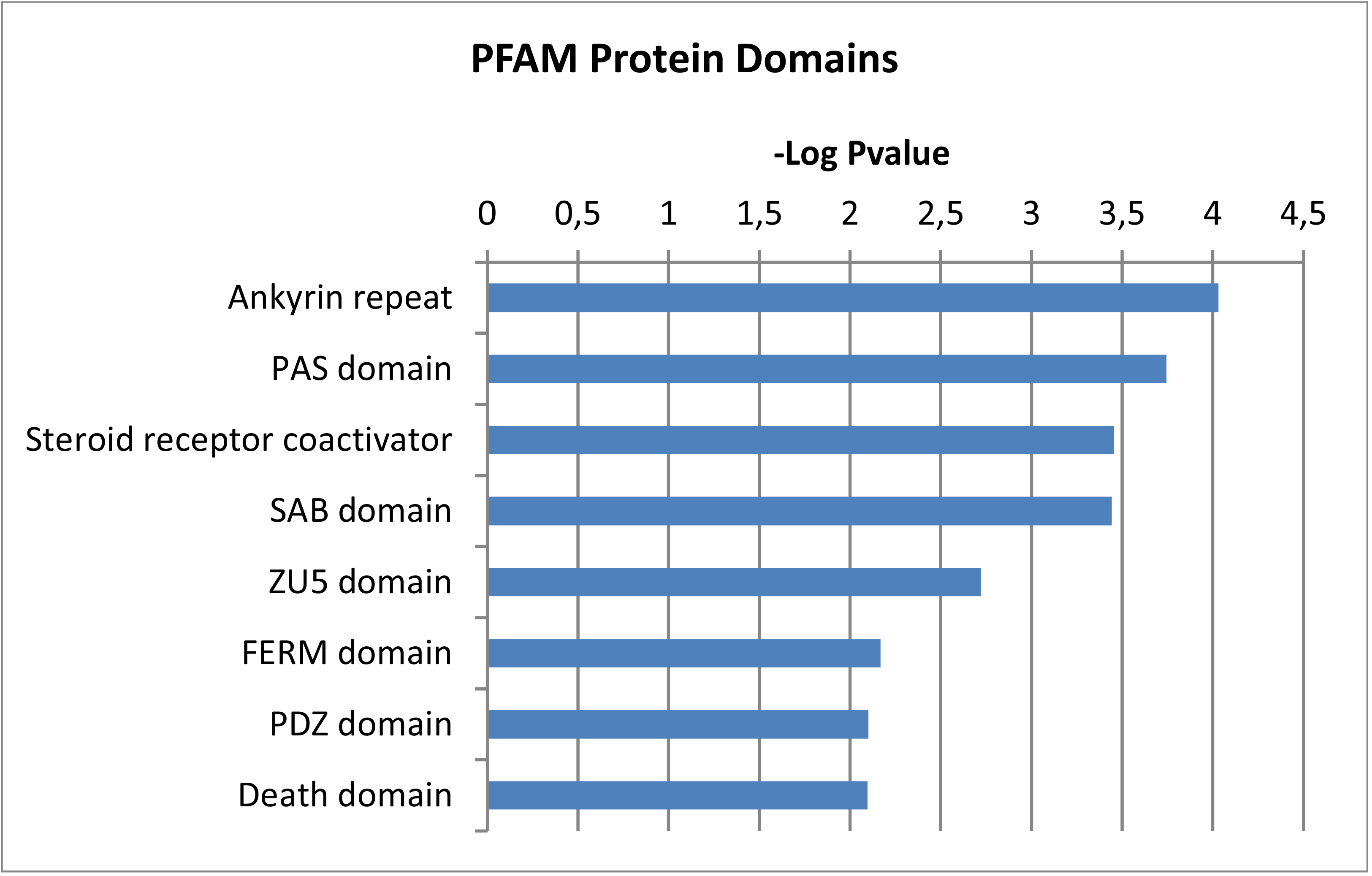


**Figure S2. PFAM protein domains significantly enriched within HAX1 binding partners obtained by Y2H.** Domains were identified using STRING web resource. The most significant group is represented by proteins with ankyrin repeats (FDR 9.33E-05), a common protein-protein interaction motifs. The other groups include PAS domain (molecular sensor), SAB and FERM domains (cytoskeletal-associated), ZU5 domain (associated with death domain and ankyrins), PDZ domain and death domain.


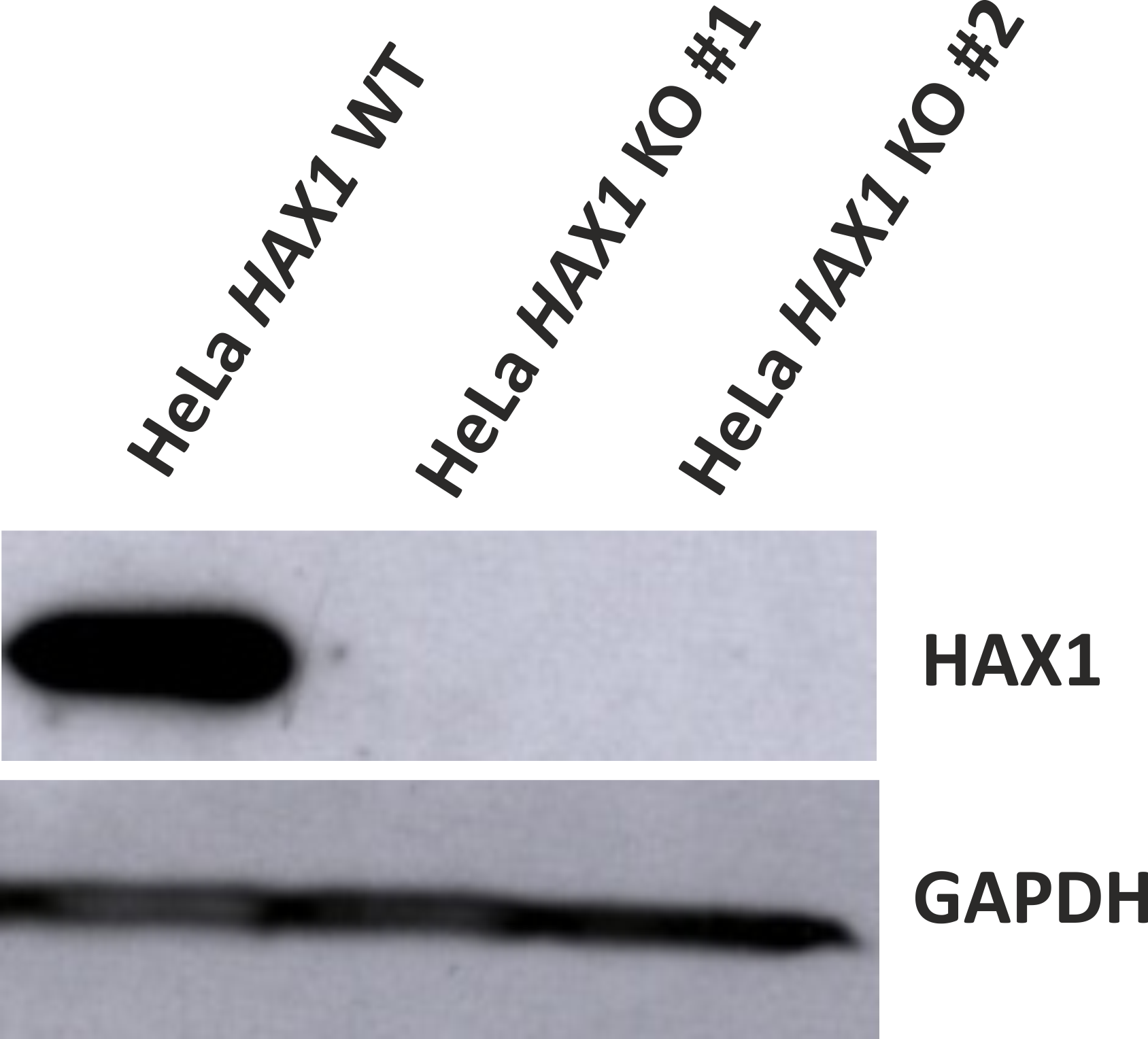


**Figure S3. *HAX1* knockout in HeLa cells.** Western blot of the two tested*HAX1* KO cell lines, *HAX1* KO #1 was used in the experiments. Reference: GAPDH.


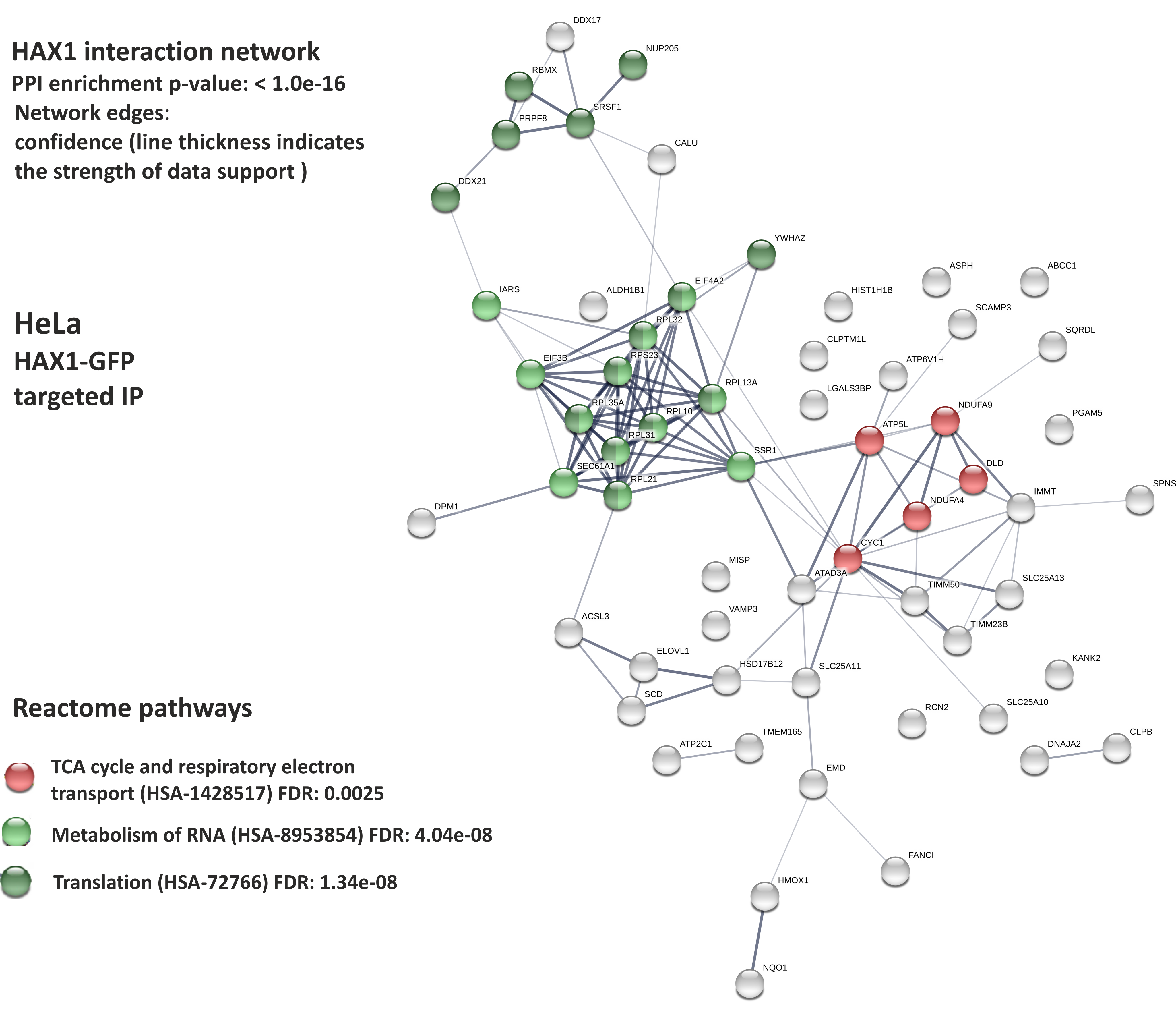


**Figure S4. STRING analysis of the HAX1-GFP interaction network in HeLa cells.** Proteins of the specified Reactome pathwayspathways (TCA cycle and respiratory electron transport, translation, RNA metabolism), forming nodes with the most number of edges are marked.


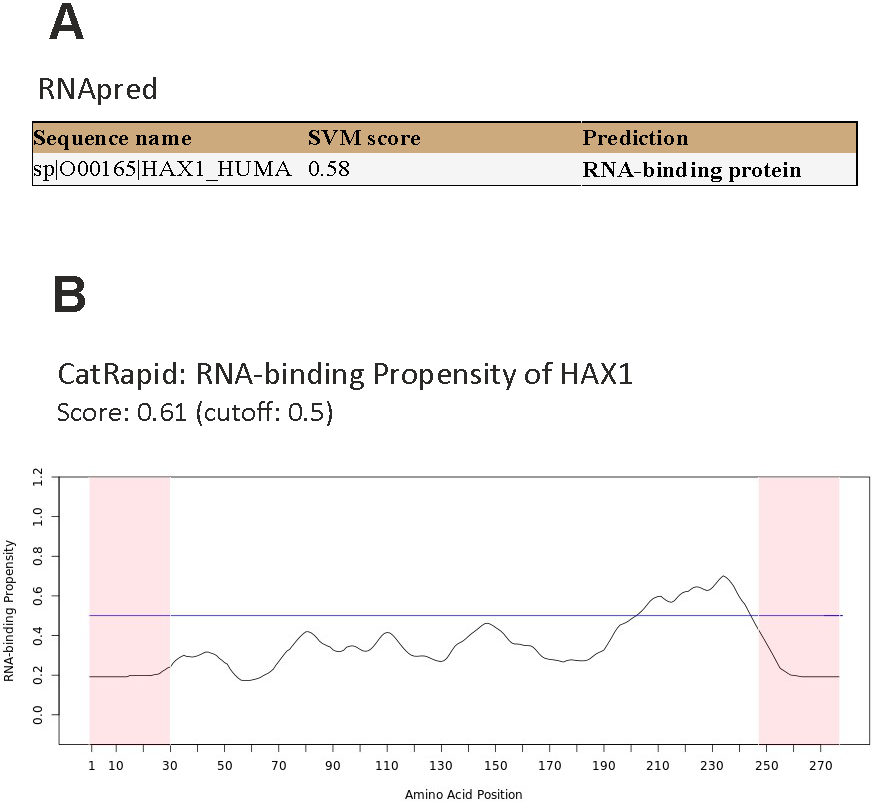


**Figure S5. HAX1 is predicted to be an RNA-binding protein by the two independent prediction algorithms.** A. RNApred: amino acid composition based SVM prediction for HAX1 B. catRapid: structural-based prediction, identifies a potential RNA-binding region within HAX1 C-terminal part (194-259 aa).


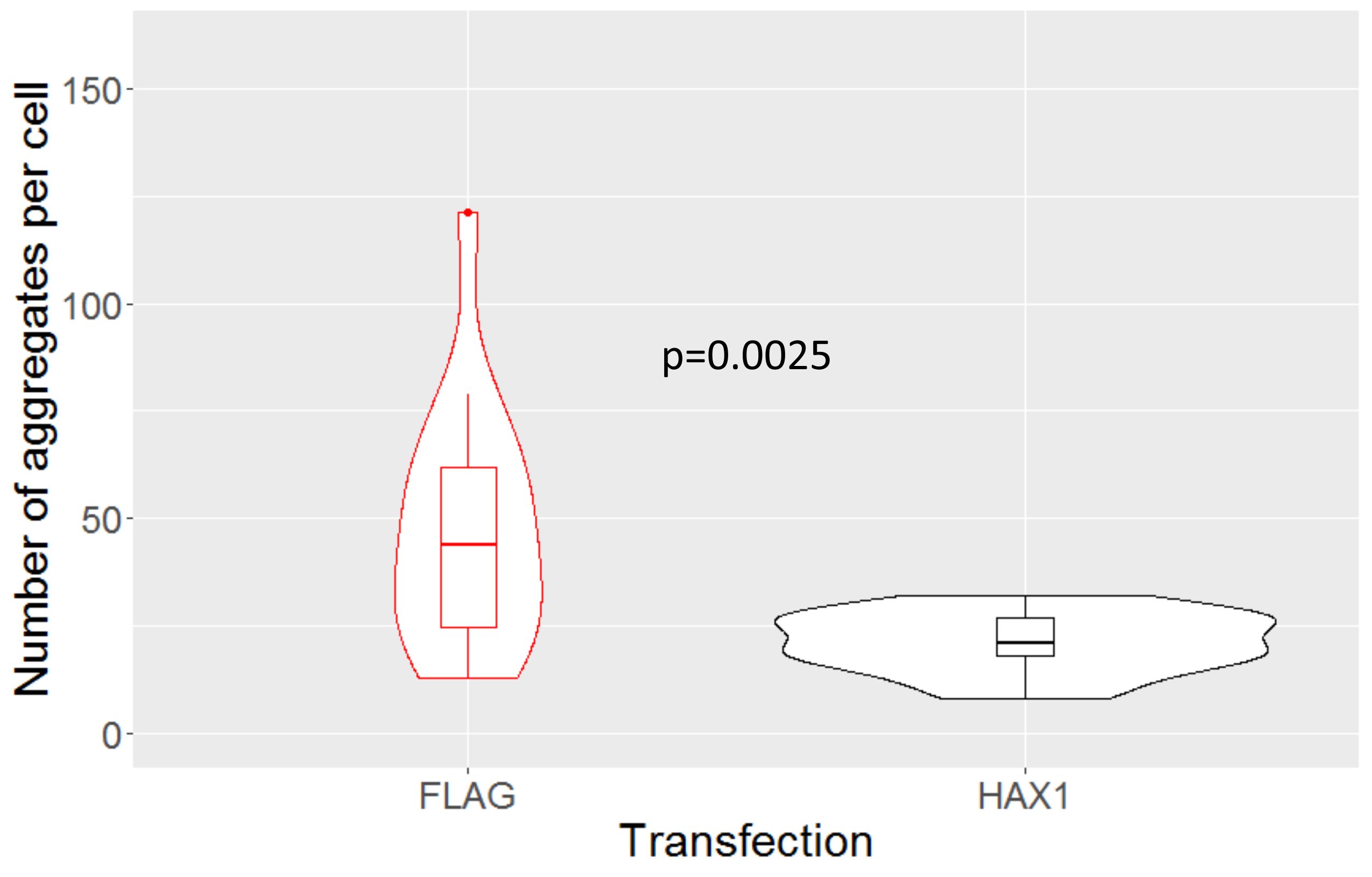


**Figure S6. Quantification of intracellular protein aggregates in WT HAX1 rescue experiment.** Aggregates were counted inHela *HAX1* KO cells transiently transfected with WT HAX1-FLAG vector (rescue experiment) and the control FLAG vector. For each cell line protein aggregates from ≈ 14-16 cells were quantified. Difference between cell lines was assessed by Mann Whitney U test (p-value=0.0025).


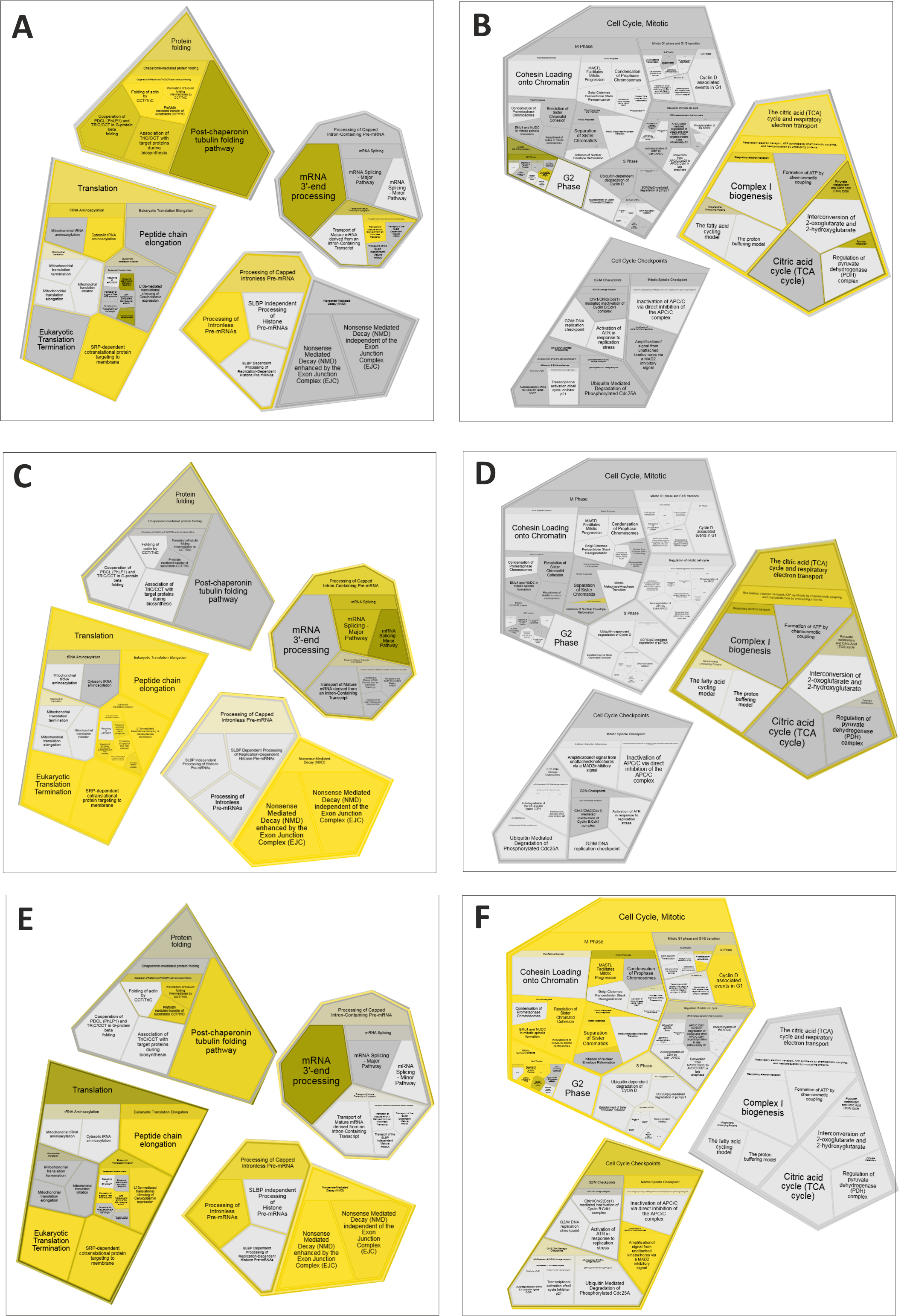


**Figure S7. HAX1 interactomes display specific pathway enrichments depending on the studied cell line.** Reactome (Voronoi Tree-maps) representation of the enrichment in the specific pathways in HeLa and MCF7 cells. A,B – HeLa (endogenous HAX1), C,D- Hela (HAX1-GFP), E,F – MCF7. While some pathways are consistently enriched (translation, protein folding, pre-mRNA processing) for both cell lines (A,C –HeLa, E- MCF7), some are cell-line specific (TCA cycle and respiratory electron transport in HeLa - B,D cell cycle in MCF7 - F).
